# Predicting the protein targets for athletic performance-enhancing substances

**DOI:** 10.1186/1758-2946-5-31

**Published:** 2013-06-25

**Authors:** Lazaros Mavridis, John BO Mitchell

**Affiliations:** 1 Biomedical Sciences Research Complex and EaStCHEM School of Chemistry, Purdie Building, University of St Andrews, North Haugh, St Andrews, Scotland KY16 9ST, UK

**Keywords:** Protein target prediction, Polypharmacology, Machine learning, Side effects, Multi-label prediction, Drugs in sport, Drug repurposing

## Abstract

**Background:**

The World Anti-Doping Agency (WADA) publishes the Prohibited List, a manually compiled international standard of substances and methods prohibited in-competition, out-of-competition and in particular sports. It would be ideal to be able to identify all substances that have one or more performance-enhancing pharmacological actions in an automated, fast and cost effective way. Here, we use experimental data derived from the ChEMBL database (~7,000,000 activity records for 1,300,000 compounds) to build a database model that takes into account both structure and experimental information, and use this database to predict both on-target and off-target interactions between these molecules and targets relevant to doping in sport.

**Results:**

The ChEMBL database was screened and eight well populated categories of activities (K_i_, K_d_, EC50, ED50, activity, potency, inhibition and IC50) were used for a rule-based filtering process to define the labels “active” or “inactive”. The “active” compounds for each of the ChEMBL families were thereby defined and these populated our bioactivity-based filtered families. A structure-based clustering step was subsequently performed in order to split families with more than one distinct chemical scaffold. This produced refined families, whose members share both a common chemical scaffold and bioactivity against a common target in ChEMBL.

**Conclusions:**

We have used the Parzen-Rosenblatt machine learning approach to test whether compounds in ChEMBL can be correctly predicted to belong to their appropriate refined families. Validation tests using the refined families gave a significant increase in predictivity compared with the filtered or with the original families. Out of 61,660 queries in our Monte Carlo cross-validation, belonging to 19,639 refined families, 41,300 (66.98%) had the parent family as the top prediction and 53,797 (87.25%) had the parent family in the top four hits. Having thus validated our approach, we used it to identify the protein targets associated with the WADA prohibited classes. For compounds where we do not have experimental data, we use their computed patterns of interaction with protein targets to make predictions of bioactivity. We hope that other groups will test these predictions experimentally in the future.

## Background

The use of performance-enhancing substances in sport, “doping”, not only jeopardizes the health of the athletes, but also threatens the integrity of sporting competition. The World Anti-Doping Agency (WADA) defines what chemical compounds and medical procedures are prohibited by publishing the prohibited list, an international standard for identifying substances and methods prohibited in-competition, out-of-competition and in particular sports. The list groups methods and substances into categories, prohibiting both specified compounds within each class and also "other substances with a similar chemical structure or similar biological effect" [1].

In previous computational work, [2,3] we demonstrated that molecules can be classified into performance-enhancing classes using MACCS and CDK cheminformatics descriptors and machine learning methods including Random Forest, k-Nearest Neighbours and Naive Bayes. We subsequently [4] introduced the UFS-MACCS hybrid descriptor, combining shape and chemistry information, using this to classify a dataset containing 5,245 molecules in ten prohibited classes from the 2005 WADA dataset and 111,231 presumed inactive molecules from the National Cancer Institute (NCI) database. These classification exercises, however, were based entirely on molecular similarity and included no explicit predictions of interactions of compounds with protein targets.

In silico protein target prediction has recently become the subject of intense research [5-12], since it helps us both to infer and to understand molecular bioactivities. The incorporation of such predictions into our workflow represents one of the main advances of this work. As well as being valuable for understanding the performance-enhancing effects of molecules, target predictions are important for in silico toxicology and early drug development. Due to the inherent promiscuity of many molecules in binding to multiple protein targets, plurality of bioactivities must be considered when building models to predict the protein targets or pharmacological activities of small organic compounds. Thus, in addition to using predictions of the primary target for in silico virtual screening, identifying secondary protein target interactions facilitates the prediction and interpretation of off-target effects [13]. Such predictions play a critical role in linking biological effects at the organism level to molecular interactions at the protein-ligand level.

Many publicly available databases, such as ChEMBL, [14] BindingDB, [15] DrugBank, [16] PubChem, [17] KiBank, [18] the PDSP Ki Database, [19] ChemProt-2.0 [20] and PDBbind [21] and commercial products including WOMBAT [22] and MDDR, [23] contain either specific assay data for the binding of compounds with targets, or associations of molecules with pharmacological properties. Databases such as ChEMBL bring together in one place bioactivity data for hundreds of thousands of different molecules and for thousands of protein targets. When combined with information from sources such as DrugBank, these can also be associated with specific biological and pharmacological activities. Those molecules which have been investigated in different assays may have activities listed against more than one target. A known limitation in molecular bioactivity data is that not every compound has been experimentally assayed against all targets, thus the matrix of available molecule-target data is sparse. Cheminformatics target prediction methods can fill in these gaps with predicted data, allowing the bioactivity spectrum of a molecule’s activity against the whole panel of targets to be assessed.

Here we propose a novel methodology that can be used to predict unexplored compound to target associations, illustrated using a number of compounds explicitly mentioned in the WADA prohibited list, by taking into account the wealth of information that is found in the ChEMBL database. Orthogonally, for each target we can identify the set or sets of mutually similar ligands that are active against it. We will also show here that special care has to be taken when one wants to use this vast amount of information, because there is a lot of noise (non-active compounds) and the data include protein targets with multiple binding sites or modes, and also some classes based on organisms rather than on proteins. These data require careful curation and handling in order to generate useful knowledge and avoid erroneous conclusions. Therefore, one of the steps of our methodology is to apply a clustering algorithm, capable of finding the optimum number of clusters for a given dataset, in order to identify structurally different groups of ligands. Diverse ligands with distinct scaffolds modulating one target may represent alternative binding modes for the same active site, but on occasion may also indicate binding at an allosteric site or a pharmacologically distinct function.

Given a prediction profile for a query compound, we can identify the pattern or patterns of activity against particular targets associated with that bioactivity. Our methodology can identify novel targets, as well as known ones, associated with a given pharmacological function. We sometimes identify just one such pattern of target interaction for a given activity or, on other occasions, find distinct sets of targets whose modulation is associated with the same function.

Our work allows early identification of potential doping molecules. These compounds can then be prioritised for experimental testing, ahead of those with negative in silico predictions. The use of this computational technology could massively reduce the need for animal or human experiments. Our results can be interpreted as a quantitative definition of the “similar chemical structure” criterion, based on similar predicted protein-target interactions, which will prevent inactive molecules being prohibited and hence protect athletes against unjust disqualification.

## Methods

### Dataset

ChEMBL currently has 8,845 families of compounds and 1,059,559 unique compounds, which are associated with the targets, based on experimental activity data derived from 44,682 publications. Each of the targets has a number of compounds associated with it. Each such association implies the existence of an experimental datum indicating activity or otherwise of the molecule against the target – however this may be that the molecule is found to be inactive. Thus, compounds can be associated with targets in ChEMBL without evidence of activity or sometimes without any experimental values. A compound can be a member of more than one family; that is, either associated with or active against more than one target.

### Filtered families

**Figure 1:**
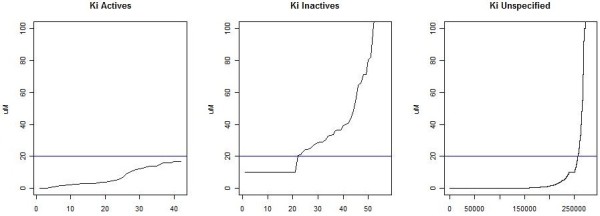
**Defining activity based on a Ki cut-off.** Experimental Ki values of active, inactive and unspecified compounds found in ChEMBL.

In order to fill in the blanks in the target-molecule bioactivity matrix, we have to predict whether a given molecule will be active against a particular target. The first step is assembling a list of other molecules in the database that are active against the target, using a definition of “active” that is valid across the different kinds of experimental data. We took the eight most common categories of affinity data (IC50, Ki, Kd, EC50, ED50, potency, activity, inhibition) relevant to bioactivity, and applied a number of rules in order to generate sets of molecules that are explicitly and experimentally determined to be bioactive, as shown in Table 1. These rules depend on the distribution of values of the relevant quantity within ChEMBL and on the ranges of values corresponding to the relatively few compounds explicitly labelled as “active” or “inactive” against that target, as shown for Ki values in Figure 1. This process generates bioactivity based definitions of families, which we refer to as filtered families.

**Table 1:** Bioactivity thresholds

**Activity**	**Active**	**Inactive**
IC50	*≤50 μM*	*>50 μM*
K_i_	*<20 μM*	*≥20 μM*
K_d_	*≤10 μM*	*>10 μM*
EC50	*≤40 μM*	*>40 μM*
ED50	*≤10 μM*	*>10 μM*
Potency	*≤10 μM*	*>10 μM*
Activity	*≥40%*	<40%
Inhibition	*≥45%*	<45%

The eight selected bioactivity categories and the rule-based thresholds that were selected.

### Refined families

**Figure 2:**
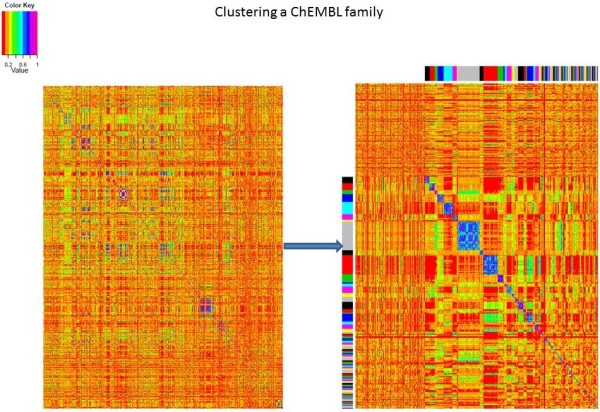
**Clustering the CHEMBL1871 family (Androgen Receptor Ligands).** The left hand frame shows the compounds in the filtered family in their original order. In the right hand frame, the multi-coloured strips show the proposed restructuring of these compounds into meaningful clusters, with each colour representing a different grouping of structurally similar compounds and hence one of the 126 separate refined families; the top left hand region is populated by singletons.

Although the filtered families consist of compounds that have significant experimental activities against the relevant targets, there are many targets that have distinct groups of ligands with different scaffolds. This may be because there is more than one binding site, or because different scaffolds can fit the same site. Figure 2 shows an example family from ChEMBL, one of the Androgen Receptor families (ChEMBL1871), with a number of different clusters of compounds. Splitting such a family into smaller groups based on ligand structure will allow us to identify the different sets of ligands; therefore PFClust [24] (brief description in Additional file 1) was applied to all the filtered ChEMBL families. We selected the PFClust algorithm because it is a parameter free clustering algorithm and does not require any kind of parameter tuning. We could instead have used any one of many well-known clustering algorithms, but then we would have had to decide on a stopping criterion for the algorithm. Since this is not straightforward, and a separate analysis on the morphology of the clusters would have been required, we decided to use our own in-house software. The compounds were clustered on the basis of their chemical structures, represented by Circular Fingerprints (CFP) [25] as discussed below. This leads to a set of refined families, each consisting of a group of molecules which share similarity of both chemical structure and bioactivity.

### Molecular fingerprints and similarities

Throughout this study, the molecules are represented as vectors of CFPs. In order to calculate the pairwise similarity between two molecules we use Tanimoto similarity scores, [26] which we transform into probabilities (p-values) using an appropriate kernel function.

**Figure 3:**
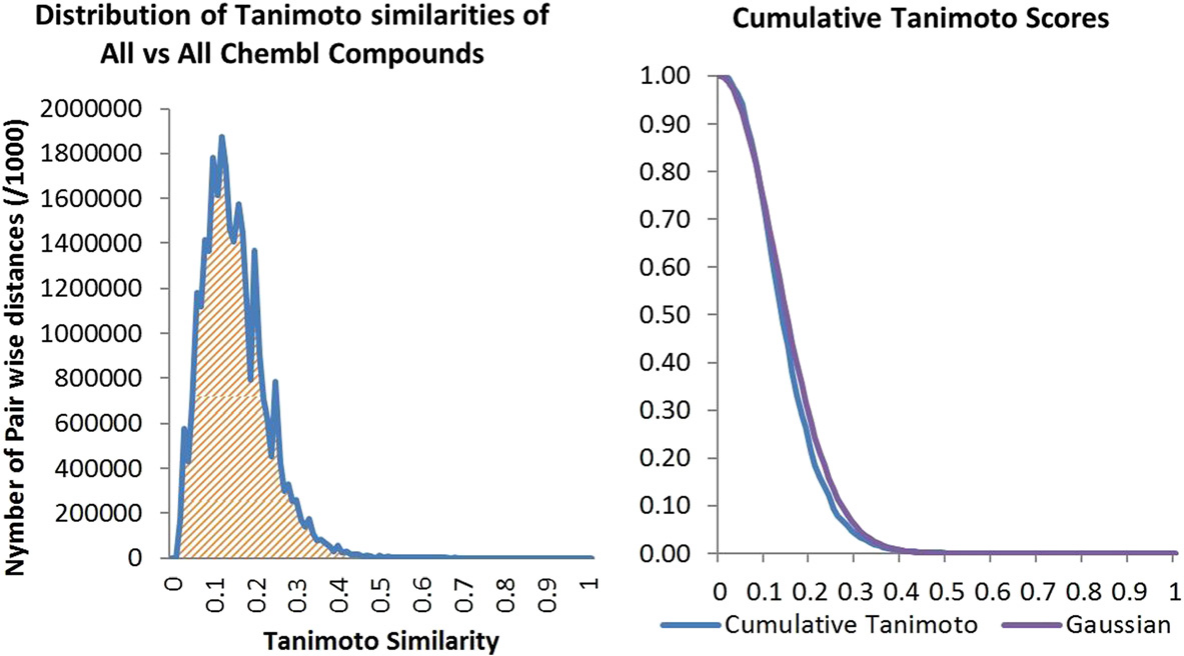
**Distributions of Tanimoto similarity scores and the fit of the data to a Gaussian.** On the left is the distribution of all pairwise Tanimoto similarities between pairs of molecules in ChEMBL version 11. The plot on the right, fitting the similarities to a Gaussian distribution, shows the proportion of molecule pairs that have similarity greater than the value on the x-axis.

In order to find the best suited kernel function for our data, we have calculated the Tanimoto similarities of all against all compounds in the ChEMBL database, the resulting distribution being shown in Figure 3. ChEMBL consists of 1,059,559 unique compounds and can be used as a representative set for estimating the distribution of Tanimoto similarities amongst all the possible subsets that we might wish to select.

We fitted a kernel probability function to our data using a Gaussian distribution, as seen in Figure 3.

### Converting a similarity into a probability (pairwise p-value)

Now we can calculate the probability that a given random pairwise similarity score X is bigger than a value x as p(X > *x*) Using the fitted Gaussian function, we can transform a Tanimoto similarity into a p-value p(X > x) as follows:

$$p\left(X>x\right)=p\left(X>t\left(x_{i},x_{j}\right)\right)=e^{-\frac{t{\left(x_{i},x_{j}\right)}^{2}}{2h^{2}}}$$

where t(x_i_,x_j_) is the Tanimoto similarity between molecules x_i_ and x_j_. We empirically found the best smoothing factor (h) to be 0.125.

### Assessing the similarity of a test molecule to a known family using the molecule-family Parzen-Rosenblatt value (PR-score)

An essential part of this work is to assign activities for molecule-target pairs that have no experimental data. Thus, we need to calculate how similar a given molecule is to a class or group of molecules. Typically this is defined by interaction with a given target in ChEMBL, or by a biological activity in a database like DrugBank. We can calculate and visualize the similarities of test compounds to a gold standard set of pre-defined families. To calculate how similar the molecule x_i_ is to the members of family *ω*, we enumerate the pairwise p-values of molecule x_i_ with all members of *ω =* {*x*_1_, *x*_2_, … , *x*_*n*_} Having calculated the distribution of *p*(*t*[*x*_*i*_,*ω*]) between molecule x_i_ and the known members of *ω*, we will apply the Parzen-Rosenblatt (PR) [27,28] kernel density estimation method to estimate the probability density function of *p*(*t*[*x*_*i*_,*ω*]). We calculate the density estimation of x_i_ given *ω* as follows:

$$f\left(x_{i},\omega \right)=\frac{1}{n}\overset{n}{\underset{j=1}{\sum }}p\left(X>t\left(x_{i},\omega_{x_{j}}\right)\right)$$

where n is the number of members of *ω* and p(*X* > (*x*_*i*_,*ω*_*xj*_)) is the p-value of x_i_ with x_j_, a typical member of *ω*.

### Validation

In order to validate our methodology, we performed a fivefold Monte Carlo cross-validation for each of the different ChEMBL family definitions: the original ChEMBL with all the compounds assigned to their label based ChEMBL families; bioactivity-based filtered families defined with the help of our rule based scheme; and finally the refined families obtained by clustering the filtered ones on chemical structure using PFClust. For each cross-validation run, we removed 10% of the members of each family, which we then used as a test set of queries. To investigate the relative performances using the three different definitions of families, we calculated two validation metrics. For computing both measures, we classified a hit to the parent family from which the query compound was taken as a true positive (TP), and hits to all other families as false positives (FP). For the first measure, we took the four top hits for each query and counted the TPs and FPs amongst these. For the second metric, we used the results of the same runs in order to calculate the Matthews Correlation Coefficient (MCC) [29], a measure of prediction success.

$$\mathrm{MCC}=\frac{\left(\mathrm{TP}\times \mathrm{TN}\right)-\left(\mathrm{FP}\times \mathrm{FN}\right)}{\sqrt{\left(\mathrm{TP}+\mathrm{FP}\right)\left(\mathrm{TP}+\mathrm{FN}\right)\left(\mathrm{TN}+\mathrm{FP}\right)\left(\mathrm{TN}+\mathrm{FN}\right)}}.$$

### Identifying the targets of the explicitly prohibited WADA molecules

We used 211 molecules that are explicitly mentioned in the WADA prohibited list (Table 2) as queries against the three versions of the families we have derived from ChEMBL: (a) original families based on ChEMBL labels; (b) filtered families based on bioactivity; (c) refined families consisting of scaffold-groups within a given filtered family.

**Table 2:** Number of molecules in each WADA prohibited class in this study

	**WADA list**
P2- Beta-Blockers	20
S1- Anabolic Agents	72
S3- Beta-2-Agonists	-
S4- Hormone Antagonists & Modulators	14
S5- Diuretics & Masking Agents	20
S6- Stimulants	64
S7- Narcotics	11
S8- Cannabinoids	10
S9- Glucocorticoids	-
Total	211

For seven WADA-defined classes of prohibited compounds and each of the three definitions of families above, we used our methodology to retrieve from ChEMBL the most significant families having p-values less than 0.05. This allows us to identify biological targets relevant to each category of performance-enhancing pharmacological activity. We check whether these molecules have explicit activities against these targets in ChEMBL, and if so we exclude them from the test for the relevant family.

We created a matrix in which the columns were the explicitly prohibited compounds in that class, the rows were the relevant families retrieved from ChEMBL, and the values were the relevant values of the Parzen-Rosenblatt function f(x_i_,ω). Each column of this matrix was considered as a vector and we calculated the pairwise Euclidean distances between the points obtained by considering these vectors as position vectors relative to the same origin. Based on these distances, we used PFClust to cluster the compounds.

### How many different groups of ligands exist in each prohibited class?

#### Clustering of compounds by structure

We have used PFClust to identify the number of structurally different groups in each WADA prohibited class. All the compounds were represented as CFP fingerprints and we calculated all against all p-values for each class, similarly to how we previously clustered structures to generate refined families from filtered families.

#### Clustering of compounds by activity

For each WADA class, we performed a database search and a vector of PR-scores against the refined families was retrieved. Using these vectors for each compound as position vectors, we calculated the Euclidean distances between the resulting points and a similarity matrix was created; we again clustered the vectors using PFClust.

## Results

### Validation

**Figure 4:**
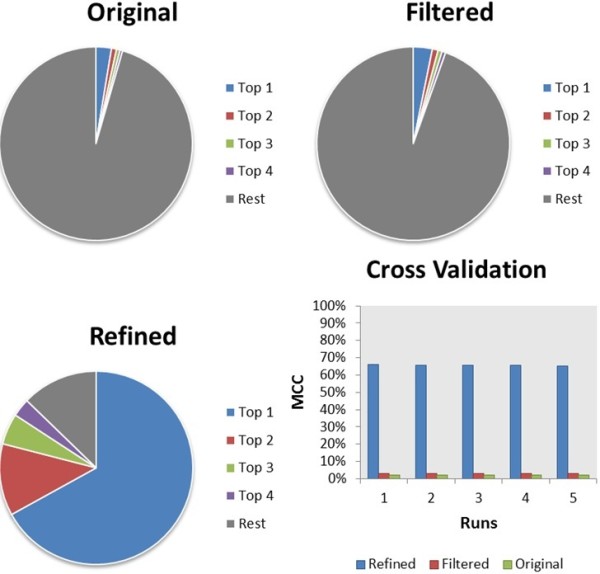
**Validation results.** Validation results for the three models of ChEMBL. On the top row we have the results for the original (left) and filtered (right) models. On the bottom left are the results for the refined model and on the bottom right we show the MCC values for each of the five runs.

The TPs and FPs obtained in the first four top-ranked positions for each query in all the cross-validation runs for each of the three definitions of families were analysed; the results are shown in Figure 4. For the original ChEMBL definition of families, we had 136,646 queries that belonged to 5,443 families, from which (on average over five runs) only 3,500 (2.58%) had the parent family as the top prediction, and only 6,078 (6.61%) had the parent family amongst the top four hits. Similarly, for the filtered families we had 78,369 queries that belonged to 3,563 families, from which on average only 2,490 (3.18%) had the parent family as the top prediction and only 4,256 (7.21%) had the parent family in the top four hits. In contrast, using the refined families gave a significant increase in predictivity. Out of 61,660 queries belonging to 19,639 families, 41,300 (66.98%) had the parent family as the top prediction and 53,797 (87.25%) had the parent family in the top four hits.

For the original label-based ChEMBL families, the MCC value of 0.02 indicates that there is no meaningful correlation between the experimental data and the predictions. We see only a very small and insignificant improvement in the MCC, from 0.02 to 0.03, on applying the rule based filtering to obtain bioactivity-based filtered families. These are disappointing results that are probably mainly influenced by the number of families that either collate all results for a given organism or tissue type, or represent proteins with multiple binding sites. The compounds that are members of these families will be quite diverse and it is then unlikely that our method will retrieve the parent family as the correct one. Due to the nature of these families, their size is significantly larger than of those of the simple protein target families representing one binding site; we observe this to be a big influence on the results.

In contrast, we see a considerable improvement when we use structural clustering to obtain refined families; the MCC for the refined bioactivity-based families from ChEMBL is 0.66. We also note that many of the predictions counted as FP nonetheless have biologically relevant connections between the predicted family and the compound, so the quoted MCC may underestimate the true number of biologically meaningful predictions.

### Identifying the targets of the explicitly prohibited WADA molecules

For each of the seven classes defined in the WADA Prohibited List, we queried the refined families from ChEMBL using every such compound as a query. For every WADA class, a heat map with the top predictions (PR-score ≤ 0.05) was calculated. Furthermore, for each class there is a table that summarizes the experimental validation for the most confident predictions, those with PR-scores below 0.05.

### P2 Beta blockers

**Figure 5:**
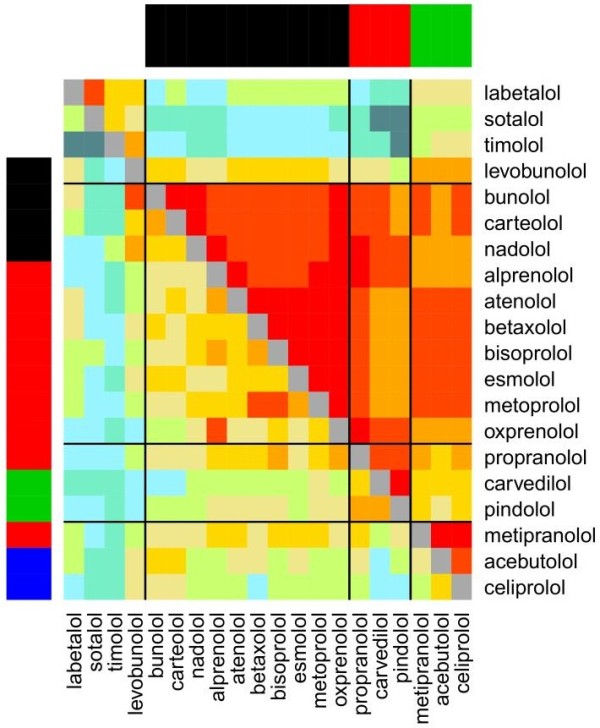
**Correspondence between activity-based and structure-based clusters for Beta blockers.** PFClust results for the P2 compounds. Activity-based clustering is shown on the x-axis, the three clusters being the black, red and green horizontal ribbons at the top of the Figure, while the activity singletons are white. Structure-based clustering is shown by the blue, red (non-contiguous), green and black vertical ribbons against the y-axis, while structural singletons are also white. The ordering of the molecules, and the division by horizontal and vertical lines, are the same on both axes and represent the bioactivity-based clustering. Coloured cells above the main diagonal represent the similarity in bioactivity between the two molecules; those below represent structural similarity.

**Figure 6:**
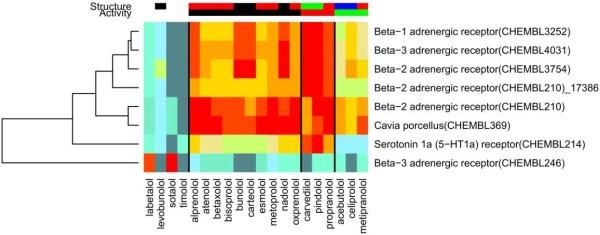
**Molecule-target associations for Beta Blockers.** The predicted molecule-target associations obtained by querying the 20 explicitly prohibited P2 beta blocker molecules against our refined families.

For the P2 class, there are 20 explicitly prohibited compounds clustered by bioactivity into three groups and four singletons (and by structure into four groups and three singletons), see Figure 5. The top ChEMBL predictions are shown in Figure 6. Every compound, except timolol and levobunolol, was predicted with a good PR-score for at least one family. We see in Table 3 that the majority of the families are Beta-1, 2 & 3 adrenergic receptor families (six out of eight). This is what we expect to find, since beta blockers are named for their well-known interactions with such beta receptors. A strong connection was shown between the Cavia porcellus family and the compounds of group one (in black). ChEMBL has a number of such families that group all experimental data for a given species, Cavia porcellus being the guinea pig. After refining these families, we see good predictions for some compounds, presumably based on chemical scaffolds alone since there is no common bioactivity. Other families also generate some interesting results, such as the serotonin 1a receptor which is indicated to make off-target interactions with pindolol [30].

**Table 3:** Beta blocker results

**Compound**	**Target**	**PR-Score**	**E-Value**
*P2-Beta Blockers*			
*Alprenolol (266195)*	*Cavia Porceullus (369)*	*0.039*	*LogB/F = −0.158*
*Carvedilol (723)*	*β-1 adrenergic receptor (3252)*	*0.032*	*Ki = 0.81 nM*
	*β-2 adrenergic receptor (210)*	*0.044*	*Ki = 0.166 nM*
	*β-2 adrenergic receptor (3754)*	*0.047*	*Prediction*
	*β-3 adrenergic receptor (4031)*	*0.036*	*Prediction*
*Pindolol (500)*	*β-1 adrenergic receptor (3252)*	*0.017*	*Ki = 1 nM*
	*β-2 adrenergic receptor (210)*	*0.015*	*Ki = 0.4 nM*
	*β-2 adrenergic receptor (3754)*	*0.026*	*Inhibition = 84%*
	*β-3 adrenergic receptor (4031)*	*0.018*	*Ki = 1 nM*
	*Serotonin 1a (5-HT1a (214)*	*0.026*	*Ki = 24 nM*
*Propranolol (27)*	*β-2 adrenergic receptor (210)*	*0.003*	*IC50 = 12 nM*
*Sotalol (471)*	*β-3 adrenergic receptor (246)*	*0.009*	*IC50 = 7200 nM*

Experimental validation of the predictions for the most significant PR-Scores (≤0.05) for the Beta Blocker P2 WADA class. Where no experimental activity exists in ChEMBL, the compound-family association retains the status of ‘Prediction’.

### S1 Anabolic agents

For the S1 family, there are 72 explicitly prohibited compounds clustered by activity into three groups and one singleton (but by structure into eight groups and 12 singletons), see Figure S1 which is provided as part of Additional file 2. The top ChEMBL predictions are also shown in Figure S1A, again part of Additional file 2. For 35 compounds we have made no prediction, given the threshold score of 0.05; for the remaining 37 compounds, there were 52 targets which were predicted for at least one compound. Table S1, in Additional file 2, shows all these 37 compounds with their predicted families. Of the 52 refined families, two were structurally distinct groups of ligands for the androgen receptor, which is known to bind testosterone. The biggest group of families with near-identical predicted profiles of interaction with S1 compounds, containing 19 members, was the GABA receptors. Previous studies have shown that anabolic steroids induce region-specific and subunit-specific rapid modulation of GABA receptors [31]. The second largest group numbers 11 families, all associated with two cytochrome P450 targets (17A1 and 19A1), both of which play an important role in the synthesis of steroid hormones (steroidogenesis) [32]. The last group of families, with six members, comprises two distinct groups of ligands for each of three β-1,3-glucuronyltransferase receptors, which have proven connections with steroids [33]. Four of the remaining families are ligands for carbonic anhydrase; drugs used for osteoporosis are potential inhibitors of this enzyme [34]. Vitamin D, having a very close relationship with the steroid hormone family, is prescribed for osteoporosis. Other interesting hits include the UDP-glucuronosyltransferase 2B7, of which androsterone is a representative substrate, [35] a species family Xenopus laevis, and even bacterial and protozoan families (Mycobacterium tuberculosis and Trypanosoma brucei). Both of these infections have clinical relationships with usage of various steroids, [36,37] with Trypanosoma brucei glucose-6-phosphate dehydrogenase being inhibited by dehydroepiandrosterone and epiandrosterone [38] and treatment with corticosteroids being found to increase susceptibility to Mycobacterium tuberculosis infection [36].

### S4 Hormone antagonists and modulators

For the S4 WADA class, there are 14 explicitly prohibited compounds clustered by activity into two groups and three singletons (and by structure into three groups and two singletons), see Figure S4 in Additional file 2. The top ChEMBL predictions are shown in Figure S4A in Additional file 2. For six of the compounds, we make no prediction with a high significance value. For the remaining eight compounds, there are 33 targets which were predicted for at least one compound. Table S4, in Additional file 2, shows all the compounds with their predicted families. Of the 33 targets, 15 were estrogen receptors (α, β and γ) and three were cytochrome P450 11A1/19A1, which are all targets with known functions relevant to the endocrine system. Four phosphodiesterase targets and the MCF7S target were predicted for both tamoxifen and toremifene, for which associations there is again significant experimental validation and supporting literature [39,40].

### S5 Diuretics and masking agents

For the S5 family, there are 20 explicitly prohibited compounds clustered by activity into four groups and three singletons (and by structure into one group and 17 singletons), see Figure S5 in Additional file 2. The top ChEMBL predictions are shown in Figure S5A in Additional file 2. For only five of the 20 compounds is there at least one high significance prediction. Table S5, also in Additional file 2, shows all the compounds with their predicted families. From the 186 targets, 20 were the carbonic anhydrases (I/II/III/IV/IX/VA/VB/VI/VII/XII/XIII/XIV) and the carbonic anhydrase-related proteins (10/2/8), all of which showed a highly significant score for acetazolamide. As can be seen from Table S5, there is very strong experimental support for each of those 20 targets with acetazolamide. We also show that three amiloride-sensitive families, as well as the epithelial cells family, had significant scores against amiloride, with strong experimental support cited in ChEMBL. The compound spironola was only predicted to interact with the androgen receptor, again with strong experimental support. On the other hand, mannitol was predicted for the maltase-glucoamylase target, probably because the target is experimentally known to interact with maltose which has a very similar chemical structure to mannitol. The remaining 159 targets were all predicted to interact strongly only with desmopressin; for five of those interactions there was experimental validation in ChEMBL.

### S6 Stimulants

For the S6 class, there are 64 explicitly prohibited compounds clustered by activity into eight groups and three singletons (and by structure into eight groups and six singletons), see Figure S6 in Additional file 2. The top ChEMBL predictions are shown in Figure S6A, also in Additional file 2. For this family, only six of the 64 compounds had at least one target predicted with high significance. Table S6, again in Additional file 2, shows the six compounds with their predicted families. There are 20 families, of which six are ryanodine receptors (RyR), a class of intracellular calcium channels primarily expressed in skeletal muscle (RyR1), myocardium (RyR2) and brain (RyR3). Another family comprises ligands for the norepinephrine transporter, a monoamine transporter for which amphetamine-like drugs are substrates [41]. We also see other interesting receptors, such as monoamine oxidase B, σ-opioid receptor, β-1 adrenergic receptor, and the glutamate NMDA receptor.

### S7 Narcotics

For the S7 class, there are eleven explicitly prohibited compounds clustered by activity into two groups and two singletons (and by structure into one group and seven singletons), see Figure S7 in Additional file 2. The top ChEMBL predictions are shown in a Figure S7A in Additional file 2. For two of the eleven compounds, there was no family predicted with high significance. Table S7, also in Additional file 2, shows the 67 predicted families and the experimental data, when available. The table shows very good agreement between predictions and experiment. Of the 67 targets, 52 are δ, κ, μ and σ opioid receptors. We also see four species target families, derived from Mus musculus and Cavia porcellus. Four glutamate NMDA receptor families are also predicted, for which we found good experimental validation in ChEMBL.

### S8 Cannabinoids

**Figure 7:**
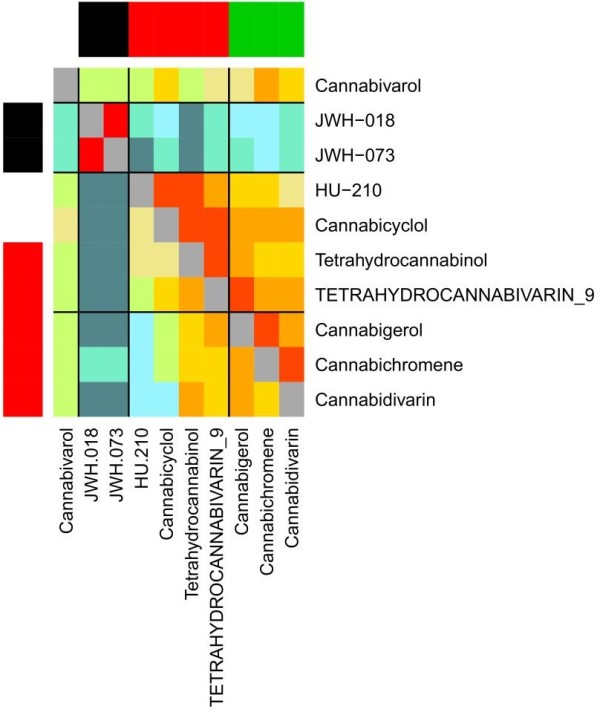
**Correspondence between activity-based and structure-based clusters for Cannabinoids.** PFClust results for the S8 compounds. Activity-based clustering is shown on the x-axis, the three clusters being the black, red and green horizontal ribbons at the top of the Figure, while the activity singletons are white. Structure-based clustering is shown by the red and black vertical ribbons against the y-axis, while structural singletons are also white. The ordering of the molecules, and the division by horizontal and vertical lines, are the same on both axes and represent the bioactivity-based clustering. Coloured cells above the main diagonal represent the similarity in bioactivity between the two molecules; those below represent structural similarity.

**Figure 8:**
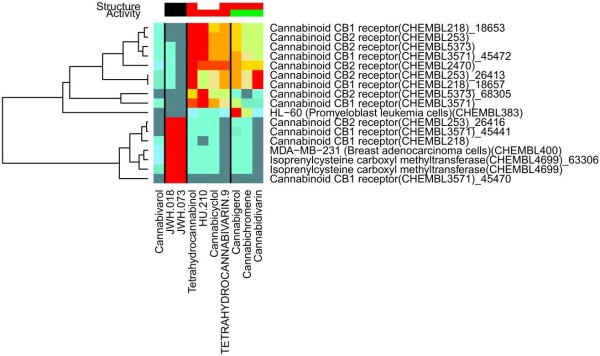
**Molecule-Target Associations for Cannabinoids.** The predicted molecule-target associations obtained by querying the 10 explicitly prohibited S8 cannabinoid molecules against our refined families derived from ChEMBL.

For the S8 class we have ten query molecules JWH-018, JWH-073, HU-210, tetrahydrocannabivarin-9, tetrahydrocannabinol, cannabicyclol, cannabigerol, cannabivarol, cannabichromene and cannabidivarin, which are split by bioactivity into three small groups and one singleton (and by structure into two groups and three singletons), see Figure 7. The top ChEMBL predictions are shown in Figure 8. There are 17 resulting refined families, of which 13 are cannabinoid CB1/2 receptors. All the compounds show strong predicted affinity to at least one cannabinoid receptor, except for tetrahydrocannabivarin-9. Table 4 shows that there is again good agreement between the PR-scores and the experimental results. For some matches, there is no available experimental evidence for or against activity in ChEMBL, and the association between the compound and family retains the status of ‘Prediction’.

**Table 4:** Cannabinoid results

**Compound**	**Target**	**PR-Score**	**E-Value**
*S8-Cannabinoids*			
*Cannabidivarin (−)*	*Cannabinoid CB1 receptor (218)*	*0.037*	*Prediction*
	*Cannabinoid CB2 receptor (253)*	*0.037*	*Prediction*
*Cannabigerol (497318)*	*HL-60 (383)*	*0.047*	*Prediction*
*HU-210 (70625)*	*Cannabinoid CB1 receptor (3571)*	*0.035*	*Ki = 0.82 nM*^*a*^
	*Cannabinoid CB2 receptor (5373)*	*0.029*	*Prediction*
*JWH-018 (561013)*	*Cannabinoid CB1 receptor (218)*	*0.002*	*pKi = 8.7*
	*Cannabinoid CB1 receptor (3571)*	*0.015*	*pKi = 8.045*
	*Cannabinoid CB2 receptor (253)*	*0.009*	*pKi = 8.2*
	*Isoprenylcysteine carboxyl methyltransferase (4699)*	*0.031*	*Prediction*
	*MDA-MB-231 (400)*	*0.030*	*Prediction*
*JWH-073 (−)*	*Cannabinoid CB1 receptor (218)*	*0.002*	*Prediction*
	*Cannabinoid CB1 receptor (3571)*	*0.025*	*Prediction*
*Tetrahydrocannabinol (465)*	*Cannabinoid CB1 receptor (218)*	*0.037*	*Ki = 2.9 nM*
	*Cannabinoid CB1 receptor (3571)*	*0.037*	*Ki = 37 nM*
	*Cannabinoid CB2 receptor (2470)*	*0.034*	*Ki = 20 nM*
	*Cannabinoid CB2 receptor (253)*	*0.033*	*Ki = 3.3 nM*
	*Cannabinoid CB2 receptor (5373)*	*0.049*	*Ki = 9.2 nM*

Experimental validation of the predictions for the most significant PR-Scores (≤0.05) for the Cannabinoid S8 WADA class. Where no experimental activity exists in ChEMBL, the compound-family association retains the status of ‘Prediction’.

^a^The experimental value is for the corresponding receptor in Homo sapiens while the PR-Score is for the receptor in Rattus norvegicus. The PR-Score for the Homo sapiens receptor was just a little above the 0.05 threshold at 0.067.

## Discussion

We have shown that, using the wealth of information in ChEMBL and our refined families, we can retrieve on- and off-target predictions for most of the explicitly mentioned molecules in the WADA prohibited list. The initial rule based filtering removes the noise from the ChEMBL families, but, as we demonstrated in the validation study, this alone is not sufficient to provide adequately good results. This is because there can be more than one structural scaffold associated with a ChEMBL family, or more than one binding site for a given receptor family. Hence, using PFClust to generate refined families significantly improves the validation results. As a consequence, for example, the beta-2 adrenergic receptor family (CHEMBL210) is predicted for two different groups of ligands, those comprising activity clusters one and two (black and red) in Figure 6. The compounds of group one have a highly significant prediction for one of the two refined families (these refined families being subfamilies of the same filtered family), with 0.0 ≤ PR-score ≤ 0.2, but not for the other refined family, with 0.2 ≤ PR-score ≤ 0.4. The opposite preference is shown by the other group of compounds. A similar case is JWH-018 which shows a highly significant prediction for the cannabinoid CB1 family (CHEMBL218), as well as for cannabidivarin, with an even bigger difference between the predictions for the subfamilies. The importance of this that JWH-018 and cannabidivarin are each matched with a specific refined subfamily, and in each case the scores for the wrong subfamily are insignificant.

As for any method, the success of our approach depends on the quality of the underlying data that are available. Our methodology tries to address the problem that, for each molecule that could be synthesised and tested, only a small fraction of its activities against different targets have been assayed. For ChEMBL families that are not well populated, or for protein targets which too few compounds are assayed against, we cannot make predictions since we do not have the required data. Hence we cannot produce any predictions for a number of the compounds that are already in the WADA prohibited list.

Our current methodology has proved that it enhances the predictive power of the CFP representations, and that the filtering and refinement of ChEMBL families enriches our results. However, the portability of our target prediction approach is as important as the quality of the results for the WADA prohibited compounds. This workflow can easily be used with different molecular representation techniques, new sets of rules, and with a different clustering algorithm (with due consideration of the stopping criterion); hence it represents a truly portable methodology.

## Experimental

The computations described in Methods were carried out on a custom-built computer using an Intel i3 processor @ 3.10Ghz with 4GB of RAM.

## Conclusions

We have presented here a novel application of a state-of-the-art protein target prediction approach to predict compound-target associations relevant to the athletic performance-enhancing properties of molecules. Further, we have shown how one of the most important and well-populated cheminformatics resources, the freely available ChEMBL database, can be decomposed into bioactivity-based refined families of ligands. Our refined families consist of separate scaffold-groups, and their use significantly improves the classification performance; full details of our refined families are given in Additional file 3. Our validations show an encouraging correspondence with independent experimental results, with 66.98% of test cases having the parent refined family as the top prediction and 87.25% having the parent refined family among the top four hits. Having thus validated our approach, we used it to identify the protein targets associated with the WADA prohibited classes. Across the seven WADA classes considered, we find a combination of expected and unexpected protein targets for their constituent molecules. Analysis of the literature, however, demonstrates that many of the non-obvious targets have biochemically or clinically validated connections with the expected bioactivities. For compounds where we do not have experimental data, we make predictions of bioactivity, seeing a number of very interesting predictions of relevant pharmacological activities for diverse compounds. These predictions are testable by future experiments.

## Competing interests

The authors have received funding from WADA. Other than this sponsorship, the authors declare no conflict of interest.

## Authors’ contributions

LM implemented the algorithm and carried out the experiments. JBOM conceived the original idea. Both authors participated in drafting the manuscript. Both authors read and approved the final manuscript.

## Supplementary Material

Additional file 1 **Outline of the PFClust clustering algorithm.** Summary of the PFClust algorithm including pseudocode. Click here for file

Additional file 2 **The results for the remaining WADA classes.** The additional tables and figures for the S1, S4, S5, S6, and S7 WADA classes in pdf format. Click here for file

Additional file 3 **Filtered and refined families.** For each of the filtered families, we list all the compounds in the filtered family and the refined families that they are members of. Click here for file
