# Supplementary material for: Predicting the protein targets for athletic performance-enhancing substances
Source: J Cheminform. 2013 Jun 25;5:31. doi: 10.1186/1758-2946-5-31 (PMC3701582; doi:10.1186/1758-2946-5-31)

PFClust: A Parameter Free Clustering Algorithm

Summary of the algorithm abstracted from:

L Mavridis, N Nath, JBO Mitchell, *BMC Bioinformatics*, **accepted**

Manuscript ID: 1120557388906353

Outline

Here we describe a partitional algorithm that uses the idea that each cluster can be represented as a non-predetermined distribution of the intra-cluster similarities of its members. The algorithm partitions a dataset into a number of clusters that share some common attributes, such as their minimum expectation value and variance of intra-cluster similarity. It is an agglomerative algorithm, meaning that it starts with separated objects and progressively joins them together to form clusters. PFClust is heuristic in the sense that it cannot be described in terms of optimising any single simply-expressed metric over the space of possible clusterings.

The clustering algorithm consists of two parts. The first part is the randomization, and the second part incorporates both the threshold selection and the actual clustering. Thus, in the first part (panel B of Figure X), 20 thresholds are estimated ($T_1, \ldots, T_{20}$) by a randomization process. In the second part (panels C & D of Figure X), each threshold is used to cluster the data, and the best threshold is selected. This whole process, incorporating both randomization and threshold selection, is carried out four times (panel E of Figure X). If the four resulting clusterings do not agree, the algorithm replaces the least successful of the four runs with a fresh attempt and repeats until convergence. Figure X shows a graphical representation of the clustering algorithm.

Pseudocode

I. Do four times:

<u>*Stage 1: Calculate D (the distribution of E[X]'s).*</u>

1. Do the specified "randomization" 1000 times:

    i. Randomly select a number of clusters *k*.

    ii. Randomly assign each data point α to a cluster *c*.

    iii. ∀ clusters *c*, calculate *E[X]* for the pairwise point-point similarities within *c* and include this value of *E[X]* in *D*.

2. For each of the ten percentiles {95.00%, 97.50%, 99.00%, 99.14%, 99.29%, 99.43%, 99.57%, 99.71%, 99.86% and 100.00%} of the distribution *D* of intra-cluster similarities, and for ten further thresholds corresponding to the second to eleventh highest values, retrieve a threshold value *T*.

    <u>*Stage 1A: Clustering*</u>

    i. While any α in the dataset remains unclustered:

        a. Join the two most similar currently unclustered elements to form a new cluster, provided criteria in b. are met.

        b. Calculate average similarity of each currently unclustered data point to the current cluster and keep adding the most similar available data point as a member as long as:

            - *E[X]* of the cluster > *T*.

            - The average similarity of the new member to the existing members of the current cluster > 0.85**T*.

    <u>*Stage 1B: Clustering Refinement*</u>

ii. $\forall \alpha \in$ any c, retrieve its average similarity with all the members of its current cluster. If this average similarity < T then:

a. If its average similarity with elements of any other cluster is more than that with the parent cluster, move the point α to this other cluster.

iii. Measure the Silhouette width, averaged over all points with singletons each contributing -1, and the Dunn Index for the final clustering for this T value.

3. Return the T value and resultant clustering with the best Silhouette width as the result of the run; in the event of a tie, use the Dunn Index to decide.

II. Repeat until Convergence:

Stage 2: Convergence (measure the Rand Index between each of the four runs)

1. If average Rand Index amongst all 6 pairs taken from the 4 clusterings $\geq$ 0.99, return the clustering with the best Silhouette width as the final result (algorithm converged).
2. If this average Rand Index < 0.99, the algorithm has not converged and the clustering with the lowest Silhouette width is discarded and we repeat *Stage 1* a single time to generate a new clustering.

Figure X -Visual representation of the algorithm

This figure provides a visual representation of the algorithm as a number of different steps (A to F).

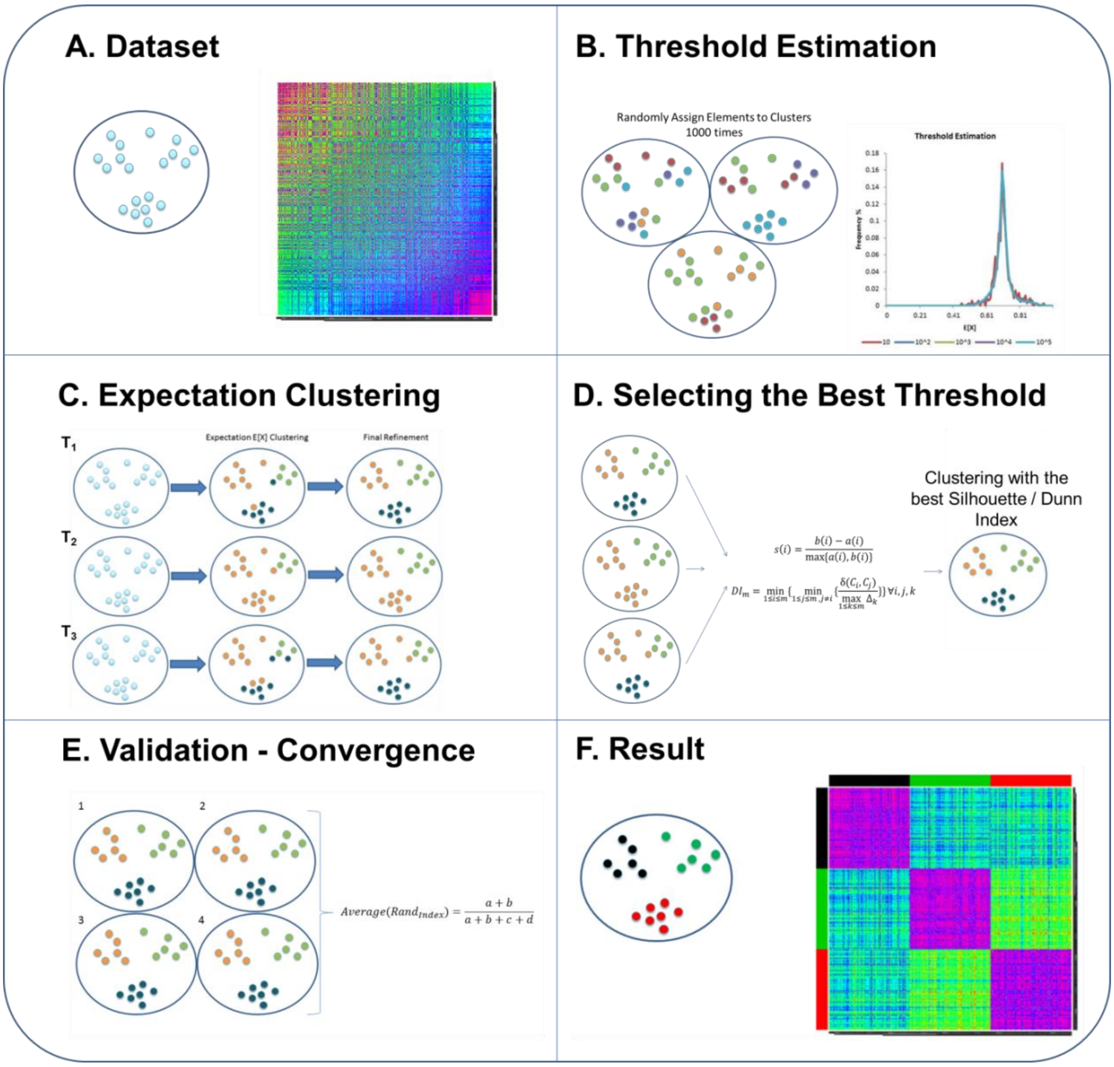

Supplement: Additional file 1 — Outline of the PFClust clustering algorithm. Summary of the PFClust algorithm including pseudocode. [file 1758-2946-5-31-S1.pdf]
