# Supplementary material for: Predicting the protein targets for athletic performance-enhancing substances
Source: J Cheminform. 2013 Jun 25;5:31. doi: 10.1186/1758-2946-5-31 (PMC3701582; doi:10.1186/1758-2946-5-31)
Supplement: Additional file 2 — The results for the remaining WADA classes. The additional tables and figures for the S1, S4, S5, S6, and S7 WADA classes in pdf format. [file 1758-2946-5-31-S2.pdf]

Compound	Target	PR-Score	E-Value
	S1-Anabolic Agents		
Androstanolon/dihydrotestosteron (27769)	*Androgen Receptor(1871)*	*0.018*	*Ki=0.2nM*
	G-protein coupled(5409)	*0.047*	*EC50=4490nM*
	HL-60(383)	*0.017*	*Prediction*
	UDP-g 2B7(4370)	*0.040*	*Activity = 18pM*
5a-androstane-3a-17a-diol(-)	*β-1,3-glucuronyltransferase-1(4067)*	*0.026*	*Prediction*
5a-androstane-3a-17b-diol(-)	*β-1,3-glucuronyltransferase-2(5209)*	*0.026*	*Prediction*
5a-androstane-3b-17a-diol(-)	*β-1,3-glucuronyltransferase-3(3958)*	*0.026*	*Prediction*
5a-androstane-3b-17b-diol(-)	*Carbonic anhydrase I(261)*	*0.033*	*Prediction*
	Carbonic anhydrase II(205)	*0.021*	*Prediction*
	Carbonic anhydrase IV(281)	*0.020*	*Prediction*
	GABA-A re; anion channel(1907607)	*0.038*	*Prediction*
	G-protein coupled bile(5409)	*0.020*	*Prediction*
	HL-60(383)	*0.004*	*Prediction*
	Human herpes virus 4(613124)	*0.045*	*Prediction*
	Lleal bile acid transporter(2778)	*0.049*	*Prediction*
	UDP-glucuronosyltransferase 2B7 (4370)	*0.018*	*Prediction*
Epiandrosterone(272196)	*β-1,3-glucuronyltransferase-1(4067)*	*0.033*	*Prediction*
	β-1,3-glucuronyltransferase-2(5209)	*0.033*	*Prediction*
	β-1,3-glucuronyltransferase-3(3958)	*0.033*	*Prediction*
	Carbonic anhydrase I(261)	*0.041*	*Prediction*
	Carbonic anhydrase IV(281)	*0.039*	*Prediction*
	GABA re-α-1 subunit(3139)	*0.017*	*Prediction*
	GABA re-α-2 subunit(2709)	*0.017*	*Prediction*
	GABA re-α-3 subunit(3387)	*0.017*	*Prediction*

	GABA re-α-4 subunit(4182)	*0.017*	*Prediction*
	GABA re-α-5 subunit(2304)	*0.017*	*Prediction*
	GABA re-α-6 subunit(4710)	*0.017*	*Prediction*
	GABA re-β-1 subunit(2839)	*0.017*	*Prediction*
	GABA re-β-3 subunit(4236)	*0.017*	*Prediction*
	GABA re-π subunit(2746)	*0.017*	*Prediction*
	GABA re-θ subunit(3249)	*0.017*	*Prediction*
	GABA-A re; anion channel(1907607)	*0.036*	*Prediction*
	G-protein coupled bile(5409)	*0.009*	*EC50=3200nM*
	LXR-α(2808)	*0.044*	*Prediction*
	Mycobacterium tuberculosis(360)	*0.030*	*Prediction*
	Xenopus laevis(613478)	*0.031*	*Prediction*
Androsterone(87285)	*β-1,3-glucuronyltransferase-1(4067)*	*0.033*	*Prediction*
	β-1,3-glucuronyltransferase-2(5209)	*0.033*	*Prediction*
	β-1,3-glucuronyltransferase-3(3958)	*0.033*	*Prediction*
	Carbonic anhydrase I(261)	*0.041*	*Not Active*[1]
	Carbonic anhydrase IV(281)	*0.039*	*Prediction*
	GABA re-α-1 subunit(3139)	*0.017*	*Prediction*
	GABA re-α-2 subunit(2709)	*0.017*	*Prediction*
	GABA re-α-3 subunit(3387)	*0.017*	*Prediction*
	GABA re-α-4 subunit(4182)	*0.017*	*Prediction*
	GABA re-α-5 subunit(2304)	*0.017*	*Prediction*
	GABA re-α-6 subunit(4710)	*0.017*	*Prediction*
	GABA re-β-1 subunit(2839)	*0.017*	*Prediction*
	GABA re-β-3 subunit(4236)	*0.017*	*Prediction*
	GABA re-π subunit(2746)	*0.017*	*Prediction*

[1] inhibition < 50% @ 10 uM and thus dose-reponse curve not measured

	GABA re-θ subunit(3249)	*0.017*	*Prediction*
	GABA-A re; anion channel(1907607)	*0.036*	*Prediction*
	G-protein coupled bile(5409)	*0.009*	*EC50=6220nM*
	LXR-α(2808)	*0.044*	*Prediction*
	Mycobacterium tuberculosis(360)	*0.030*	*Prediction*
	Xenopus laevis(613478)	*0.031*	*EC50=3380nM*
1-androstenediol (-)	*LXR-alpha(2808)*	*0.039*	*Prediction*
3a-17a-androst-5-ene-3-17-diol	*Mycobacterium tuberculosis(360)*	*0.041*	*Prediction*
Androst-5-ene-3b-17a-diol	*Trypanosoma brucei(612348)*	*0.030*	*Prediction*
4 androstene-3a-17b-diol(-)	*G-protein coupled (5409)*	*0.047*	*Prediction*
4androstenediol(195836)			
Boldenone(-)	*Matrix-metalloproteinase (332)*	*0.048*	*Prediction*
Testosterone(386630)	*Androgen Receptor(1871)*	*0.044*	*Ki=29nM*
	Muscarinic acetylcholine(276)	*0.035*	*Prediction*
19 norandrosterone(-)	*Xenopus Laevis(613478)*	*0.0489*	*Prediction*
3a_5a_3_hydroxyestran_17_one			
Prasterone(90693)	*Mycobacterium tuberculosis(360)*	*0.031*	*Prediction*
Androstenedione	*CytochromeP450 19A1(1978)*	*0.031*	*IC50=300nM*
(274826)			*Ki=20nM*
17a-methyl-1-testosterone(-)	*Androgen Receptor(1871)*	*0.033*	*Prediction*
5_androstenedione(-)	*Cytochrome P450 19A1(3859)*	*0.028*	*Prediction*
	CytochromeP450 19A1(1978)	*0.002*	*Prediction*
5a-androst-1-en-3-17-diol	*Cytochrome P450 17A1(3522)*	*0.009*	*Prediction*
Androst-5-ene-3-17-diol-3a-17b	*CytochromeP450 17A1(4430)*	*0.047*	*Prediction*
(-)	*CytochromeP450 19A1(1978)*	*0.012*	*Prediction*
Norandrostenedione(-)	*Cytochrome P450 17A1(3522)*	*0.038*	*Prediction*
	CytochromeP450 19A1(1978)	*0.001*	*Prediction*

	CytochromeP450 19A1(3859)	*0.015*	*Prediction*
Methandriol(-)	*Cytochrome P450 17A1(3522)*	*0.018*	*Prediction*
	CytochromeP450 19A1(1978)	*0.038*	*Prediction*
19 nortestosterone(757)	*Cytochrome P450 17A1(3522)*	*0.003*	*Not Active*[2]
Epitestosterone(196228)	*Cytochrome P450 17A1(3522)*	*0.002*	*Prediction*
	CytochromeP450 19A1(1978)	*0.029*	*Prediction*
Clostebol(-)	*Cytochrome P450 17A1(3522)*	*0.013*	*Prediction*
	CytochromeP450 19A1(1978)	*0.037*	*Prediction*
4 hydroxy testosterone(-)	*Cytochrome P450 17A1(3522)*	*0.013*	*Prediction*
	CytochromeP450 19A1(1978)	*0.037*	*Prediction*
17 methyltestosterone (1395)	*Cytochrome P450 17A1(3522)*	*0.022*	*Prediction*
	CytochromeP450 19A1(1978)	*0.034*	*Prediction*
Calusterone(455706)	*Cytochrome P450 17A1(3522)*	*0.013*	*Prediction*
Bolasterone(-)	*Cytochrome P450 17A1(3522)*	*0.013*	*Prediction*
Oxymesterone(-)	*Cytochrome P450 17A1(3522)*	*0.021*	*Prediction*
Flyoxymesterone(1445)	*Cytochrome P450 17A1(3522)*	*0.047*	*Prediction*

Table S1. Experimental validation of the predictions for the most significant PR-Scores (≤0.05) for the S1 Anabolic Agents family. Where no experimental activity exists in ChEMBL, the association between the compound and family retains the status of ‘*Prediction*’.

[2] Inhibition < 50% @ 10 µM and thus dose-response curve not measured

Compound	Target	PR-Score	E-Value
S4-Hormones Antagonists and Modulators			
Aminoglutethimide(488)	*Cytochrome P450 11A1(4813)*	*>0.001*	*Inhibition=85%*
	Cytochrome P450 19A1(1978)	*0.032*	*Ki=90nM*
	Muscarine acetylcholine(1907609)	*0.049*	*Prediction*
Anastrozole(1399)	*Cytochrome P450 19A1(1978)*	*0.043*	*Ki=0.13nM*
Raloxifene(81)	*Estrogen Receptor α(206)*	*0.002*	*Ki=0.4nM*
	Estrogen Receptor β(242)	*0.003*	*Ki=10nM*
	Ishikawa(614649)	*0.017*	*EC50=0.04nM*
Tamoxifen(83)	*Estrogen Receptor α(3065)*	*0.039*	*Inhibition=100%*
	Estrogen Receptor β(242)	*0.006*	*IC50=170nM*
	Estrogen Receptor β(2995)	*0.039*	*Inhibition=100%*
	Estrogen-related Receptor γ(4245)	*0.000*	*Prediction*
	MCF7S(614328)	*0.036*	*IC50=7200nM*
	Phosphodiesterase 1A(3421)	*0.000*	*IC50=6750nM*
	Phosphodiesterase 1B(4425)	*0.000*	*IC50=6750nM*
	Phosphodiesterase 1C(4619)	*0.000*	*IC50=6750nM*
	Phosphodiesterase 4A(3333)	*0.000*	*IC50=6800nM*
Toremifene(1655)	*Estrogen Receptor β(242)*	*0.025*	*Prediction*
	Estrogen-related Receptor γ(4245)	*0.000*	*Prediction*
	MCF7S(614328)	*0.045*	*Prediction*
	Phosphodiesterase 1A(3421)	*0.013*	*Prediction*
	Phosphodiesterase 1B(4425)	*0.013*	*Prediction*
	Phosphodiesterase 1C(4619)	*0.013*	*Prediction*
	Phosphodiesterase 4A(3333)	*0.013*	*Prediction*
Clomifene(1200667)	*Estrogen Receptor β(242)*	*0.024*	*Prediction*
	MCF7S(614328)	*0.012*	*Prediction*

	MVLN(614185)	*0.027*	*Prediction*
Cyclofenil(141305)	*Estrogen Receptor α(3065)*	*0.019*	*Prediction*
	Estrogen Receptor β(2995)	*0.019*	*Prediction*
Fulvestrant(1358)	*Estradiol 17- β-dehydrogenase(3181)*	*0.017*	*Prediction*
	Estrogen Receptor α(206)	*0.039*	*Ki=1.04nM*
	Estrogen Receptor β(242)	*0.018*	*Ki=0.755nM*
	Ishikawa(614649)	*0.008*	*Prediction*
	MDA-MB-231(400)	*0.019*	*T/C=95%*
	Steryl-sulfatase(3559)	*0.021*	*Prediction*
	Tubulin α chain(4262)	*0.041*	*Prediction*
	Tubulin α-1-chain(3752)	*0.045*	*Prediction*
	Tubulin β chain(3394)	*0.013*	*Prediction*

Table S4. Experimental validation of the predictions for the most significant PR-Scores (≤0.05) for the S4 Hormone Antagonists and Modulators family. Where no experimental activity exists in ChEMBL, the association between the compound and family retains the status of '*Prediction*'.

Compound	Target	PR-Score	E-Value
	S5 - Diuretics and Masking Agents		
Acetazolamide(20)	*Carbonic anhydrase I(261)*	*0.009*	*Ki=260nM*
	Carbonic anhydrase II(205)	*0.012*	*Ki=12nM*
	Carbonic anhydrase III(2885)	*0.020*	*IC50=20nM*
	Carbonic anhydrase IV(281)	*0.019*	*Ki=70nM*
	Carbonic anhydrase IV(3729)	*0.018*	*Ki=74nM*
	Carbonic anhydrase IX(3594)	*0.009*	*Ki=25nM*
	Carbonic anhydrase VA(4789)	*0.010*	*Ki=60nM*
	Carbonic anhydrase VB(3969)	*0.017*	*Ki=54nM*
	Carbonic anhydrase VI(3025)	*0.019*	*Ki=11nM*
	Carbonic anhydrase VII(2326)	*0.017*	*Ki=0.8nM*
	Carbonic anhydrase XII(3242)	*0.010*	*Ki=6.7nM*
	Carbonic anhydrase XIII(3912)	*0.019*	*Ki=5.7nM*
	Carbonic anhydrase XIV(3510)	*0.019*	*Ki=41nM*
	Carbonic anhydrase-related protein 10(4645)	*0.021*	*Ki=0.8nM*
	Carbonic anhydrase-related protein 2(2420)	*0.021*	*Ki=0.8nM*
	Carbonic anhydrase-related protein 8(2410)	*0.021*	*Ki=0.8nM*
Amiloride(945)	*Amiloride-sensitive cation channel 3(5368)*	*0.005*	*IC50=4400nM*
	Amiloride-sensitive sodium channel α(1791)	*0.002*	*IC50=775nM*
	Epithelial cells(614488)	*0.003*	*Activity=96%*
Spironolactone(1393)	*Adrogen Receptor(1871)*	*0.042*	*Ki=39.4nM*
Mannitol(689)	*Maltase-glucoamylase(2074)*	*0.049*	*Prediction*
Desmopressin(376685)	*40S ribosomal protein SA(6119)*	*0.007*	*Prediction*
	Adenylate cyclase type II(2958)	*0.024*	*Prediction*
	Adenylate cyclase type III(4864)	*0.024*	*Prediction*
	Adenylate cyclase type IV(2879)	*0.024*	*Prediction*
	Adenylate cyclase type V(2880)	*0.024*	*Prediction*

	Adenylate cyclase type VI(2975)	*0.024*	*Prediction*
	Adenylate cyclase type VII(3940)	*0.024*	*Prediction*
	Adenylate cyclase type VIII(3082)	*0.024*	*Prediction*
	Angiotensin II type 2 (AT-2) receptor(257)	*0.039*	*Prediction*
	Anolis carolinensis(612688)	*0.022*	*Prediction*
	Apoptosis regulator Bcl-2(4860)	*0.042*	*Prediction*
	Apoptosis regulator Bcl-X(4625)	*0.020*	*Prediction*
	ATP-binding cassette sub-family(1641361)	*0.006*	*Prediction*
	Atrial natriuretic peptide receptor A(1988)	*0.021*	*Prediction*
	Atrial natriuretic peptide receptor B(1795)	*0.027*	*Prediction*
	Atrial natriuretic peptide receptor B(2156)	*0.007*	*Prediction*
	Atrial natriuretic peptide receptor C(2247)	*0.006*	*Prediction*
	Atrial natriuretic peptide receptor C(4711)	*0.007*	*Prediction*
	Bradykinin B1 receptor(4613)	*0.030*	*Prediction*
	Bradykinin B2 receptor(2501)	*0.017*	*Prediction*
	Brain adenylate cyclase 1(2679)	*0.024*	*Prediction*
	C5a anaphylatoxin chemotactic receptor(2373)	*0.026*	*Prediction*
	Calcitonin gene-related peptide 1(5293)	*0.041*	*Prediction*
	Calcitonin gene-related peptide 1 receptor(3798)	*0.045*	*Prediction*
	Calcitonin receptor(1832)	*0.043*	*Prediction*
	Calcitonin receptor(2204)	*0.039*	*Prediction*
	Canis familiaris(373)	*0.039*	*Prediction*
	Cavia porcellus(369)	*0.015*	*Prediction*
	CHO (Ovarian cells)(613853)	*0.045*	*Prediction*
	Coagulation factor VII(3991)	*0.009*	*Prediction*
	Coagulation factor X(244)	*0.043*	*Prediction*
	Complement C3(4917)	*0.035*	*Prediction*
	Corticotropin releasing factor receptor 1(2446)	*0.014*	*Prediction*
	Corticotropin releasing factor receptor 2(2253)	*0.016*	*Prediction*

	Corticotropin releasing factor receptor 2(4069)	*0.043*	*Prediction*
	C-X-C chemokine receptor type 4(2107)	*0.006*	*Prediction*
	Delta opioid receptor(236)	*0.011*	*Prediction*
	Delta opioid receptor(269)	*0.025*	*Prediction*
	Delta opioid receptor(3222)	*0.004*	*Prediction*
	DNA polymerase kappa(5365)	*0.018*	*Prediction*
	Fibronectin receptor alpha(3955)	*0.010*	*Prediction*
	Fibronectin receptor beta(1905)	*0.010*	*Prediction*
	Galanin receptor 1(4894)	*0.033*	*Prediction*
	Galanin receptor 2(3176)	*0.033*	*Prediction*
	Glucagon receptor(4720)	*0.027*	*Prediction*
	Gonadotropin-releasing hormone receptor(1855)	*0.007*	*Prediction*
	Gonadotropin-releasing hormone receptor(3066)	*0.006*	*Prediction*
	Growth hormone-releasing receptor(3709)	*0.020*	*Prediction*
	Homo sapiens(372)	*0.011*	*Prediction*
	Human herpes virus 1(377)	*0.045*	*Prediction*
	IgG receptor FcRn large subunit p51(5966)	*0.009*	*Prediction*
	Influenza A virus (A/PR/8/34(H1N1))(612610)	*0.035*	*Prediction*
	Influenza A virus(613740)	*0.033*	*Prediction*
	Integrin alpha-IIb(212)	*0.010*	*Prediction*
	Integrin β-3(207)	*0.010*	*Prediction*
	Integrin β-5(2600)	*0.000*	*Prediction*
	Interleukin-8(2157)	*0.019*	*Prediction*
	κ opioid receptor(237)	*0.009*	*Prediction*
	κ opioid receptor(3614)	*0.029*	*Prediction*
	κ opioid receptor(3952)	*0.008*	*Prediction*
	κ opioid receptor(4329)	*0.029*	*Prediction*
	Matriptase(3018)	*0.004*	*Prediction*
	Melanocortin receptor 1(3795)	*0.012*	*Prediction*

	Melanocortin receptor 1(4077)	*0.001*	*Prediction*
	Melanocortin receptor 3(4644)	*0.035*	*Prediction*
	Melanocortin receptor 3(4774)	*0.044*	*Prediction*
	Melanocortin receptor 4(259)	*0.033*	*Prediction*
	Melanocortin receptor 4(3719)	*0.003*	*Prediction*
	Melanocortin receptor 5(4489)	*0.044*	*Prediction*
	Melanocortin receptor 5(4608)	*0.016*	*Prediction*
	Metastin receptor(5413)	*0.032*	*Prediction*
	MT4 (Lymphocytes)(388)	*0.012*	*Prediction*
	μ opioid receptor(233)	*0.007*	*Prediction*
	μ opioid receptor(270)	*0.015*	*Prediction*
	μ opioid receptor(2858)	*0.004*	*Prediction*
	μ opioid receptor(4354)	*0.003*	*Prediction*
	Mus musculus(375)	*0.020*	*Prediction*
	Myosin light chain kinase, smooth muscle(3062)	*0.033*	*Prediction*
	Neurokinin 1 receptor(3942)	*0.045*	*Prediction*
	Neurokinin 1 receptor(4027)	*0.015*	*Prediction*
	Neuronal acetylcholine r protein α7 (2492)	*0.027*	*Prediction*
	Neuronal acetylcholine r protein β2 (1883)	*0.034*	*Prediction*
	Neuronal acetylcholine receptor s- α-3(3068)	*0.034*	*Prediction*
	Neuronal acetylcholine receptor α3/β2(1907587)	*0.025*	*Prediction*
	Neuropeptide FF receptor 1(4571)	*0.040*	*Prediction*
	Neuropeptide FF receptor 2(3425)	*0.017*	*Prediction*
	Neuropeptide S receptor(5497)	*0.021*	*Prediction*
	Neuropeptide Y receptor type 1(4777)	*0.011*	*Prediction*
	Neuropeptide Y receptor type 2(3309)	*0.014*	*Prediction*
	Neuropeptide Y receptor type 2(4018)	*0.011*	*Prediction*
	Neuropeptide Y receptor type 4(4877)	*0.008*	*Prediction*
	Neuropeptide Y receptor type 5(2548)	*0.014*	*Prediction*

	Neuropeptide Y receptor type 5(4561)	*0.009*	*Prediction*
	Neurotensin receptor 2(2580)	*0.027*	*Prediction*
	Nociceptin receptor(2014)	*0.017*	*Prediction*
	Nociceptin receptor(3621)	*0.014*	*Prediction*
	Nociceptin receptor(4503)	*0.022*	*Prediction*
	Orexin receptor 1(5113)	*0.049*	*Prediction*
	Oryctolagus cuniculus(374)	*0.041*	*Prediction*
	Oxytocin receptor(2049)	*0.000*	*Activity=63umg^{-1}*
	Oxytocin receptor(3996)	*0.001*	*Ki=0.97nM*
	Pituitary adenylate cyclase-activating (5399)	*0.034*	*Prediction*
	Plasma kallikrein(2000)	*0.006*	*Prediction*
	Serine/threonine-protein kinase AKT(4282)	*0.008*	*Prediction*
	Serpin H1(1163113)	*0.017*	*Prediction*
	Staphylococcus aureus(352)	*0.041*	*Prediction*
	Thrombin(204)	*0.004*	*Prediction*
	Thrombin(4471)	*0.048*	*Prediction*
	Thyrotropin-releasing hormone receptor 2(3875)	*0.042*	*Prediction*
	Tissue-type plasminogen activator(1873)	*0.050*	*Prediction*
	Transcription factor E2F1(4382)	*0.041*	*Prediction*
	Trypsin I(3769)	*0.028*	*Prediction*
	Type-1A angiotensin II receptor(329)	*0.023*	*Prediction*
	Type-1B angiotensin II receptor(263)	*0.021*	*Prediction*
	Tyrosine-protein kinase SRC(267)	*0.024*	*Prediction*
	Vasopressin V1a receptor(1889)	*0.001*	*Prediction*
	Vasopressin V1a receptor(2868)	*0.010*	*Ki=10.8nM*
	Vasopressin V1b receptor(1921)	*0.000*	*Prediction*
	Vasopressin V1b receptor(2659)	*0.000*	*Ki=0.2nM*
	Vasopressin V2 receptor(1790)	*0.006*	*Prediction*
	Vasopressin V2 receptor(3766)	*0.000*	*Ki=0.8nM*

	Vasopressin V2 receptor(3944)	*0.017*	*Prediction*
	Vitronectin receptor α(3660)	*0.000*	*Prediction*

Table S5. Experimental validation of the predictions for the most significant PR-Scores (≤0.05) for the S5 Diuretics and Masking Agents class. Where no experimental activity exists in ChEMBL, the association between the compound and family retains the status of '*Prediction*'.

Compound	Target	PR-Score	E-Value
	S6- Stimulants		
Fencamine(-)	*Hypoxia-inducible factor 1α(4261)*	*0.018*	*Prediction*
	Monoamine oxidase B(2039)	*0.033*	*Prediction*
Fenetyllin(-)	*Monoamine oxidase B(2039)*	*0.022*	*Prediction*
	PC-12(612556)	*0.015*	*Prediction*
	Relaxin receptor 1(1293316)	*0.036*	*Prediction*
	Ryanodine receptor 1(2727)	*0.015*	*Prediction*
	Ryanodine receptor 2(3388)	*0.015*	*Prediction*
	Ryanodine receptor 3(3370)	*0.015*	*Prediction*
	Transient receptor(6007)	*0.034*	Prediction
Methamphetamine(1201201)	*Sigma opioid receptor(287)*	*0.048*	*Ki=8320nM*
Norfenfluramine(250881)	*Neurokinin 1 receptor(249)*	*0.032*	*Prediction*
Adrenaline(679)	*β-1 adrenergic receptor(3252)*	*0.042*	*Ki=1100nM*
	Hypoxia-inducible factor 1α(4261)	*0.046*	*Potency=1uM*
	Nuclear factor NF-κ-Bp105(3251)	*0.049*	*Potency=1.7uM*
Cathine(-)	*Glutamate NMDA receptor(1907608)*	*0.025*	*Prediction*
Ephedrine(211456)	*Glutamate NMDA receptor(1907608)*	*0.032*	*Prediction*
Etamivan(-)	*Monoamine oxidase B(2039)*	*0.039*	*Prediction*
	Oryctolagus cuniculus(374)	*0.044*	*Prediction*
Levomethamphetamine(-)	*Sigma opioid receptor(287)*	*0.048*	*Prediction*
Parahydroxyamphetamine(1546)	*Norepinephrine transporter(304)*	*0.035*	*Prediction*
Pseudoephedrine(1590)	*Glutamate NMDA receptor(1907608)*	*0.032*	*Prediction*

Table S6. Experimental validation of the predictions for the most significant PR-Scores (≤0.05) for the S6 Stimulants class. Where no experimental activity exists in ChEMBL, the association between the compound and family retains the status of '*Prediction*'.

Compound	Target	PR-Score	E-Value
	S7-Narcotics		
Buprenorphine(1201894)	*δ opioid receptor (236)*	*0.017*	*Ki=1.6nM*
	κ opioid receptor (237)	*0.003*	*Ki=0.18nM*
	κ opioid receptor (3952)	*0.033*	*Ki=1.5nM*
	κ opioid receptor (4329)	*0.021*	*Prediction*
	µ opioid receptor (233)	*0.016*	*Ki=1.5nM*
	µ opioid receptor (270)	*0.034*	*IC50=0.41nM*
Diacetylmorphine(459324)	*δ opioid receptor (236)*	*0.000*	*Prediction*
	κ opioid receptor (237)	*0.000*	*Prediction*
	µ opioid receptor (233)	*0.006*	*Prediction*
	µ opioid receptor (4354)	*0.014*	*Prediction*
	Nociceptin receptor(2014)	*0.015*	*Prediction*
Fentanyl(596)	*Cavia Porcellus(369)*	*0.049*	*IC50=3.45nM*
	δ opioid receptor (236)	*0.005*	*IC50=187.4nM*
	δ opioid receptor (269)	*0.019*	*Ki=2.16nM*
	κ opioid receptor (237)	*0.014*	*Ki=196.5nM*
	Leishmania donovani(367)	*0.029*	*IC50=N/D*
	µ opioid receptor (233)	*0.013*	*Ki=3.97nM*
	µ opioid receptor (270)	*0.021*	*Ki=1.5nM*
	Plasmodium falciparum(612856)	*0.027*	*Prediction*
	Trypanosoma brucei(612348)	*0.020*	*IC50=N/D*
Morphine(70)	*Cavia porcellus(369)*	*0.041*	*IC50=68.2nM*[3]
	δ opioid receptor (236)	*0.000*	*Ki=456nM*
	δ opioid receptor (269)	*0.027*	*Ki=6nM*

[3] Opioid agonistic activity measured in guinea pig ileum

	κ opioid receptor (237)	*0.000*	*Ki=33.7mM*
	μ opioid receptor (233)	*0.002*	*IC50=65nM*
	μ opioid receptor (270)	*0.014*	*Ki=1.8nM*
	μ opioid receptor (4354)	*0.012*	*Ki=33nM*
	Nociceptin receptor(2014)	*0.013*	*Ki=13.9nM*
Pentazocine(560)	*δ opioid receptor (269)*	*0.033*	*Prediction*
	δ opioid receptor (3222)	*0.004*	*Prediction*
	Glutamate NMDA 3B(282)	*0.000*	*Prediction*
	Glutamate NMDA 2(311)	*0.024*	*Ki=842nM*
	Glutamate NMDA 3(401)	*0.041*	*Ki=842nM*
	Glutamate NMDA 4(303)	*0.005*	*Ki=842nM*
	κ opioid receptor (3952)	*0.010*	*Prediction*
	κ opioid receptor (4329)	*0.024*	*Prediction*
	μ opioid receptor (270)	*0.019*	*Ki=13nM*
	μ opioid receptor (2858)	*0.015*	*Prediction*
	σ 2 receptor(613288)	*0.009*	*IC50=29nM*
	σ opioid receptor (287)	*0.050*	*Ki=36.5nM*
	σ opioid receptor (3465)	*0.004*	*Prediction*
Hydromorphone(398707)	*Acetylcholinesterase(4078)*	*0.038*	*Prediction*
	Cavia porcellus(369)	*0.038*	*Prediction*
	δ opioid receptor (236)	*0.017*	*Ki=38nM*
	δ opioid receptor (269)	*0.022*	*Prediction*
	δ opioid receptor (3222)	*0.027*	*Prediction*
	κ opioid receptor (237)	*0.020*	*Ki=2.8nM*
	κ opioid receptor (3952)	*0.019*	*Prediction*
	μ opioid receptor (233)	*0.026*	*Ki=0.28nM*
	μ opioid receptor (270)	*0.036*	*Prediction*

	μ opioid receptor (4354)	*0.014*	*Prediction*
	Mus Musculus(375)	*0.020*	*ED50=0.08*[4]
	Nociceptin receptor(2014)	*0.036*	*Prediction*
	σ opioid receptor (3602)	*0.043*	*Prediction*
Oxycodone(656)	*δ opioid receptor (236)*	*0.018*	*Prediction*
	δ opioid receptor (269)	*0.006*	*Ki=1087nM*
	δ opioid receptor (3222)	*0.033*	*Prediction*
	κ opioid receptor (237)	*0.011*	*Prediction*
	κ opioid receptor (3952)	*0.022*	*Ki>10000nM*
	μ opioid receptor (233)	*0.015*	*Prediction*
	μ opioid receptor (270)	*0.012*	*Ki=43.6nM*
	μ opioid receptor (4354)	*0.026*	*Prediction*
	Mus Musculus(375)	*0.018*	*ED=1.5*[5]
Oxymorphone(963)	*Cavia porcellus(369)*	*0.038*	*IC50=24nM*
	Cytochrome P450 2D6(289)	*0.041*	*Prediction*
	δ opioid receptor (236)	*0.006*	*Ki=50nM*
	δ opioid receptor (269)	*0.008*	*Ki=80.5nM*
	δ opioid receptor (3222)	*0.016*	*Ki=730nM*
	κ opioid receptor (237)	*0.003*	*IC50=90nM*
	κ opioid receptor (3614)	*0.043*	*Ki=95nM*
	κ opioid receptor (3952)	*0.007*	*Ki=61.6nM*
	μ opioid receptor (233)	*0.003*	*Ki=0.78nM*
	μ opioid receptor (270)	*0.013*	*Ki=0.97nM*
	μ opioid receptor (2858)	*0.033*	*Prediction*

[4] μmol kg^{-1}

[5] μmol.kg^{-1} Narcotic agonistic activity in acetic acid mouse writhing assay after subcutaneous administration of the drug

	μ opioid receptor (4354)	*0.006*	*Ki=15nM*
	Mus Musculus(375)	*0.020*	*ED50=24nM*[6]
	σ opioid receptor (3602)	*0.033*	*IC50=0.013nM*
Pethidine(607)	*Acetylcholinesterase(4078)*	*0.032*	*Prediction*
	δ opioid receptor (236)	*0.030*	*IC50=500nM*
	δ opioid receptor (269)	*0.028*	*IC50=6000*
	κ opioid receptor (237)	*0.025*	*IC50=500nM*
	κ opioid receptor (3952)	*0.041*	*Prediction*
	μ opioid receptor (233)	*0.024*	*Ki=451nM*
	μ opioid receptor (270)	*0.040*	*IC50=6000*
	μ opioid receptor (4354)	*0.042*	*Prediction*
	Mus Musculus(375)	*0.019*	*ED50=22*[7]
	Nociceptin receptor(2014)	*0.042*	*Prediction*

Table S7. Experimental validation of the predictions for the most significant PR-Scores (≤0.05) for the S7 Narcotics class. Where no experimental activity exists in ChEMBL, the association between the compound and family retains the status of '*Prediction*'.

[6] Analgesic potency (antinociceptive activity), administered icv at peak time of 10 min in male Swiss-Webster mice

[7] $mg.kg^{-1}$ Tested for analgesic activity using tail flick assay in mice

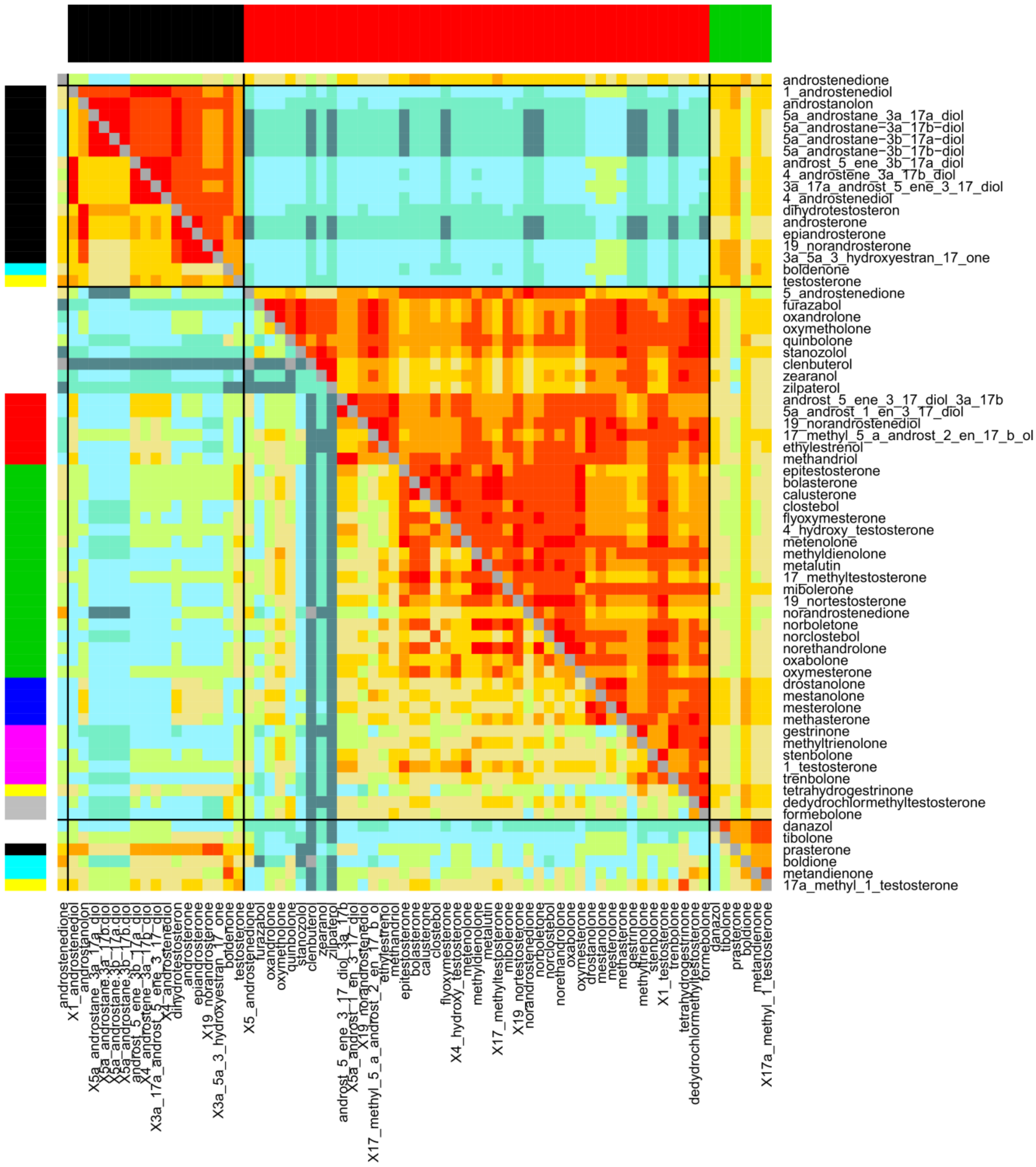

Figure S1. PFClust results for the S1 Anabolic Agents class. Activity-based clustering is shown on the x-axis, the three clusters being the black, red and green horizontal ribbons at the top of the Figure, while the activity singletons are white. Structure-based clustering is shown by the various coloured, and sometimes non-contiguous, vertical ribbons against the y-axis, while structural singletons are also white. The ordering of the molecules, and the division by horizontal and vertical lines, are the same on both axes and represent the bioactivity-based clustering. Coloured cells above the main diagonal represent the similarity in bioactivity between the two molecules; those below represent structural similarity.

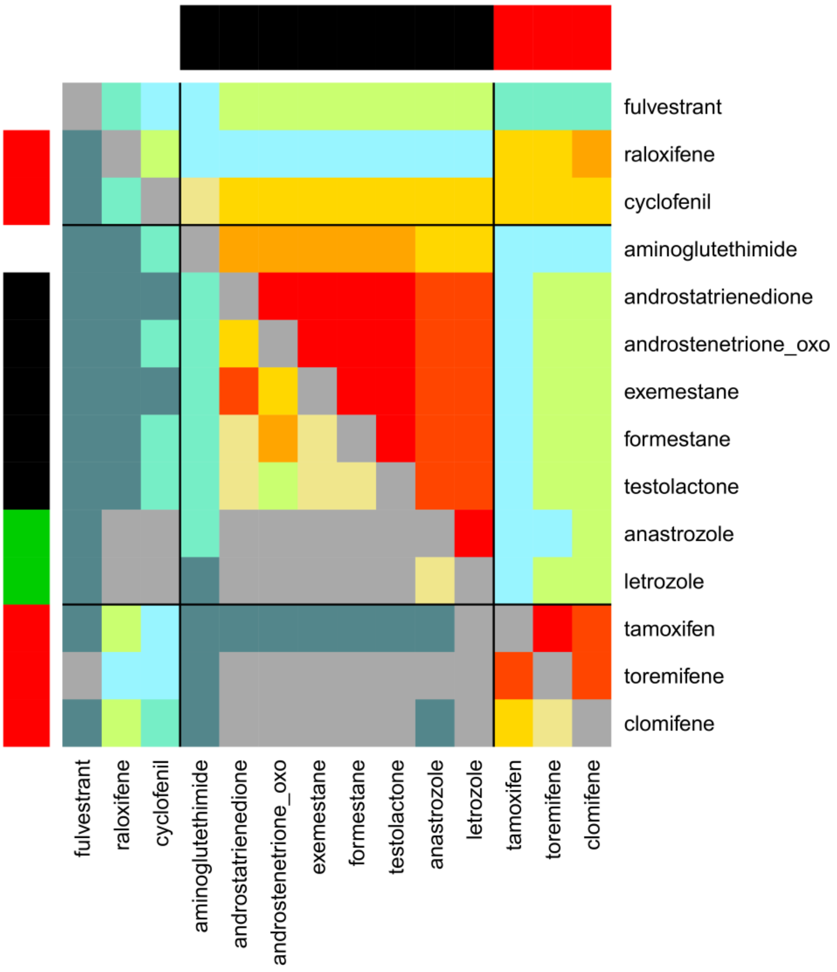

Figure S4. PFClust results for the S4 Hormone Antagonists and Modulators. Activity-based clustering is shown on the x-axis, the two clusters being the black and red horizontal ribbons at the top of the Figure, while the activity singletons are white. Structure-based clustering is shown by the red (non-contiguous), green and black vertical ribbons against the y-axis, while structural singletons are also white. The ordering of the molecules, and the division by horizontal and vertical lines, are the same on both axes and represent the bioactivity-based clustering. Coloured cells above the main diagonal represent the similarity in bioactivity between the two molecules; those below represent structural similarity.

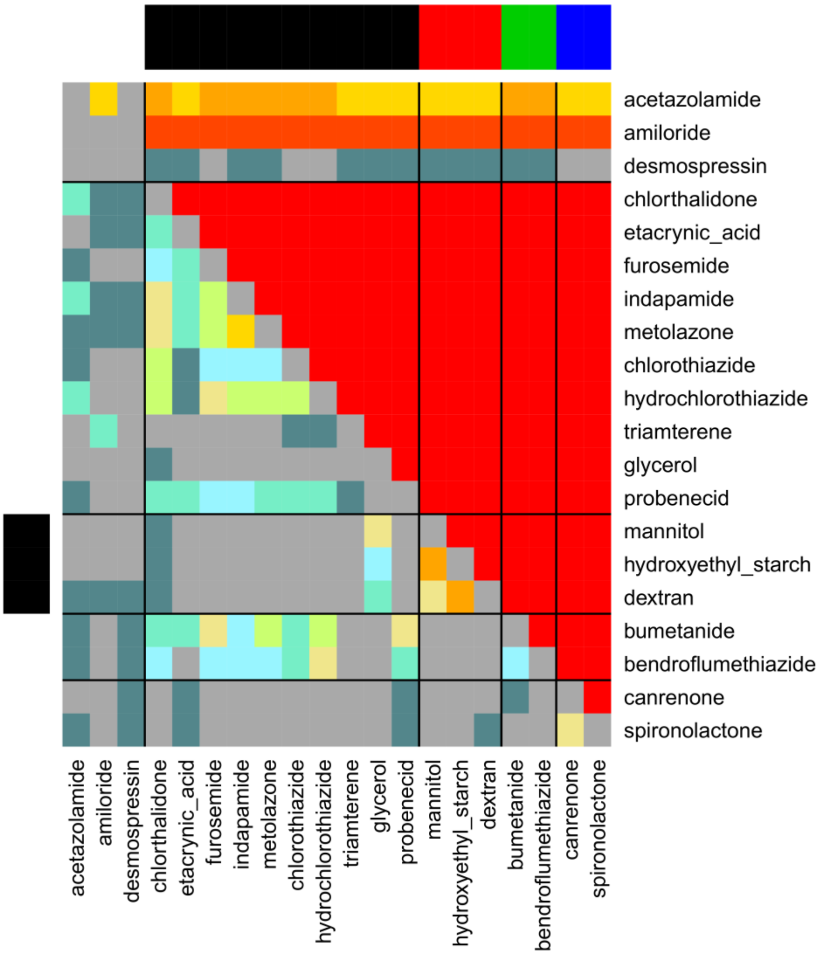

Figure S5. PFClust results for the S5 Diuretics and Other Masking Agents. Activity-based clustering is shown on the x-axis, the two clusters being the black and red horizontal ribbons at the top of the Figure, while the activity singletons are white. Structure-based clustering is shown by the red (non-contiguous), green and black vertical ribbons against the y-axis, while structural singletons are also white. The ordering of the molecules, and the division by horizontal and vertical lines, are the same on both axes and represent the bioactivity-based clustering. Coloured cells above the main diagonal represent the similarity in bioactivity between the two molecules; those below represent structural similarity.

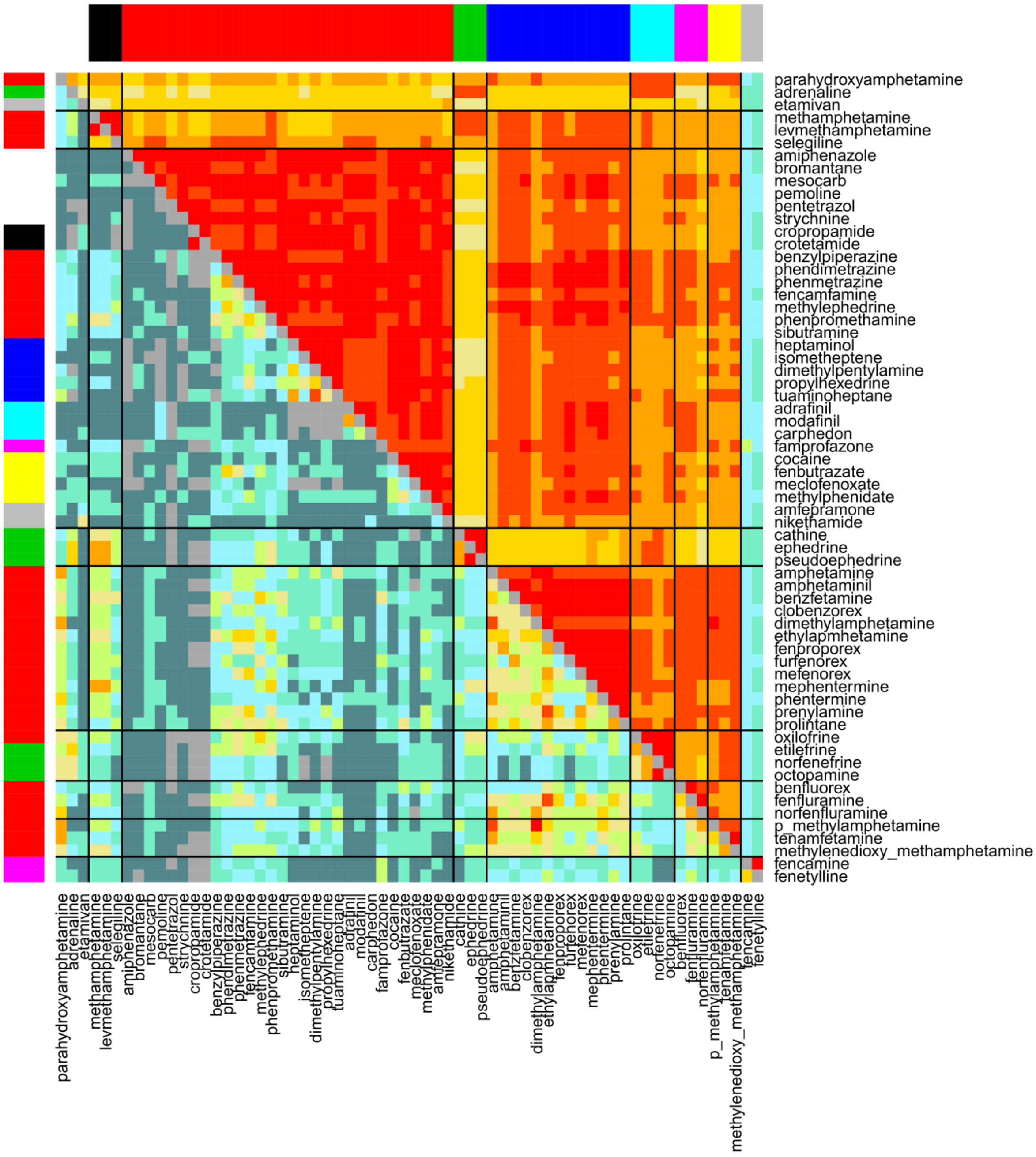

Figure S6. PFClust results for the S6 Stimulants. Activity-based clustering is shown on the x-axis, the eight clusters being the coloured horizontal ribbons at the top of the Figure, while the activity singletons are white. Structure-based clustering is shown by the various coloured, and sometimes non-contiguous, vertical ribbons against the y-axis, while structural singletons are also white. The ordering of the molecules, and the division by horizontal and vertical lines, are the same on both axes and represent the bioactivity-based clustering. Coloured cells above the main diagonal represent the similarity in bioactivity between the two molecules; those below represent structural similarity.

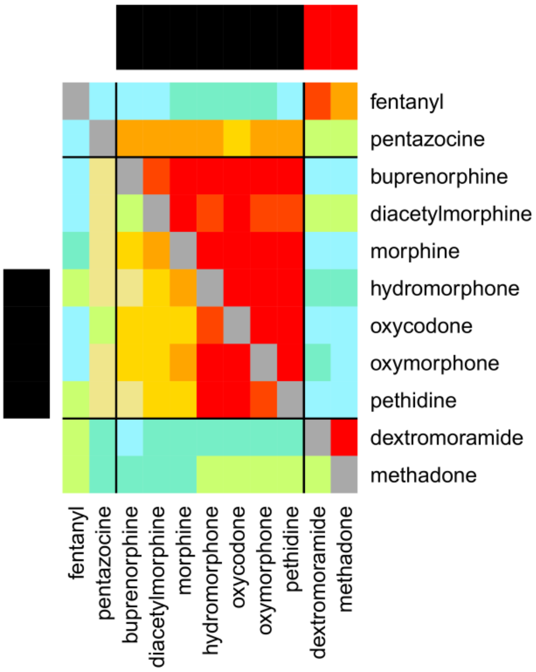

Figure S7. PFClust results for the S7 Narcotics family. Activity-based clustering is shown on the x-axis, the two clusters being the black and red horizontal ribbons at the top of the Figure, while the activity singletons are white. Structure-based clustering is shown by the black vertical ribbon against the y-axis, the remaining compounds being structural singletons which are also coloured white. The ordering of the molecules, and the division by horizontal and vertical lines, are the same on both axes and represent the bioactivity-based clustering. Coloured cells above the main diagonal represent the similarity in bioactivity between the two molecules; those below represent structural similarity.

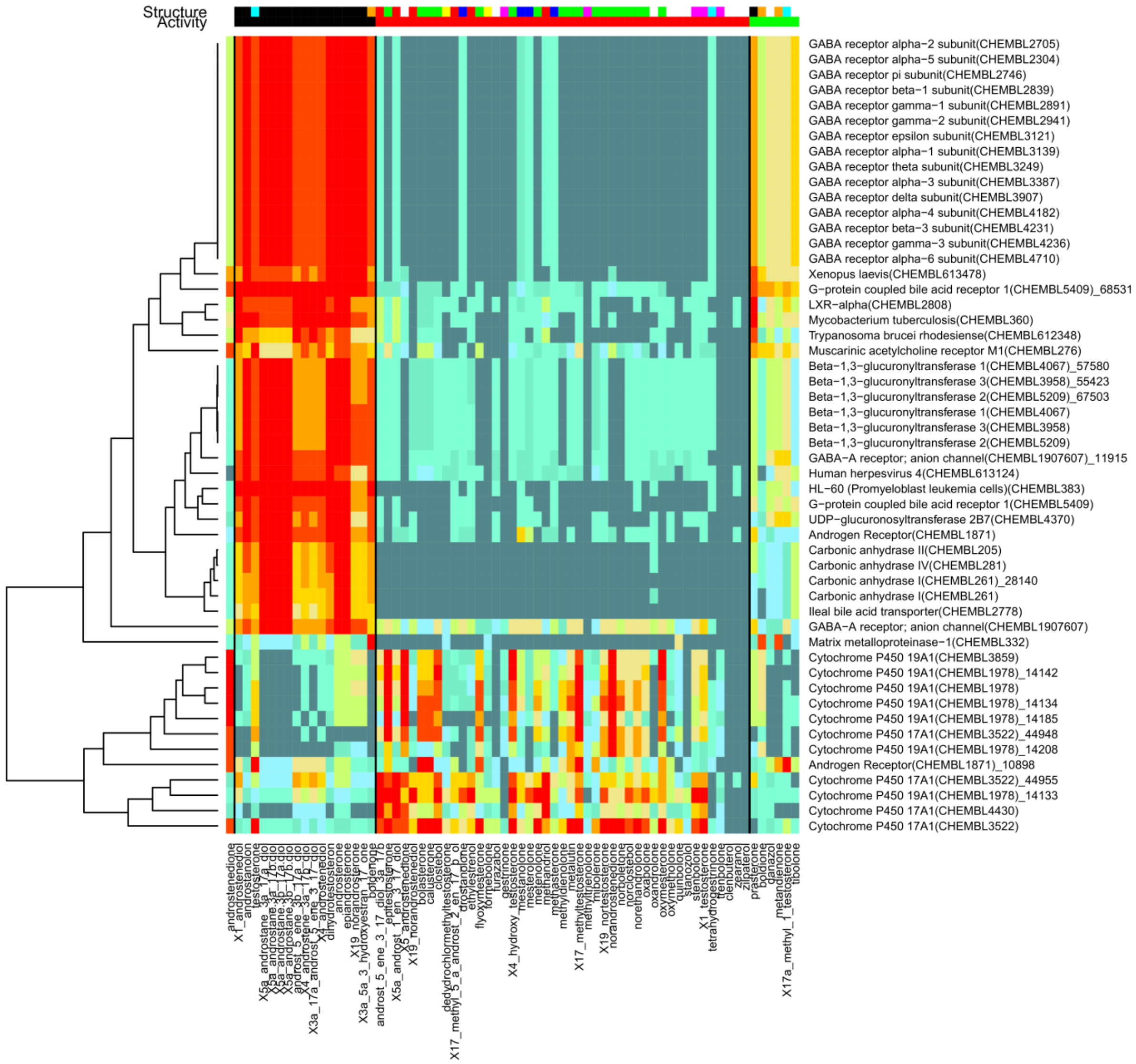

Figure S1A. The predicted molecule-target associations obtained by querying the 11 explicitly prohibited S1 Anabolic Agents against our refined families derived from ChEMBL.

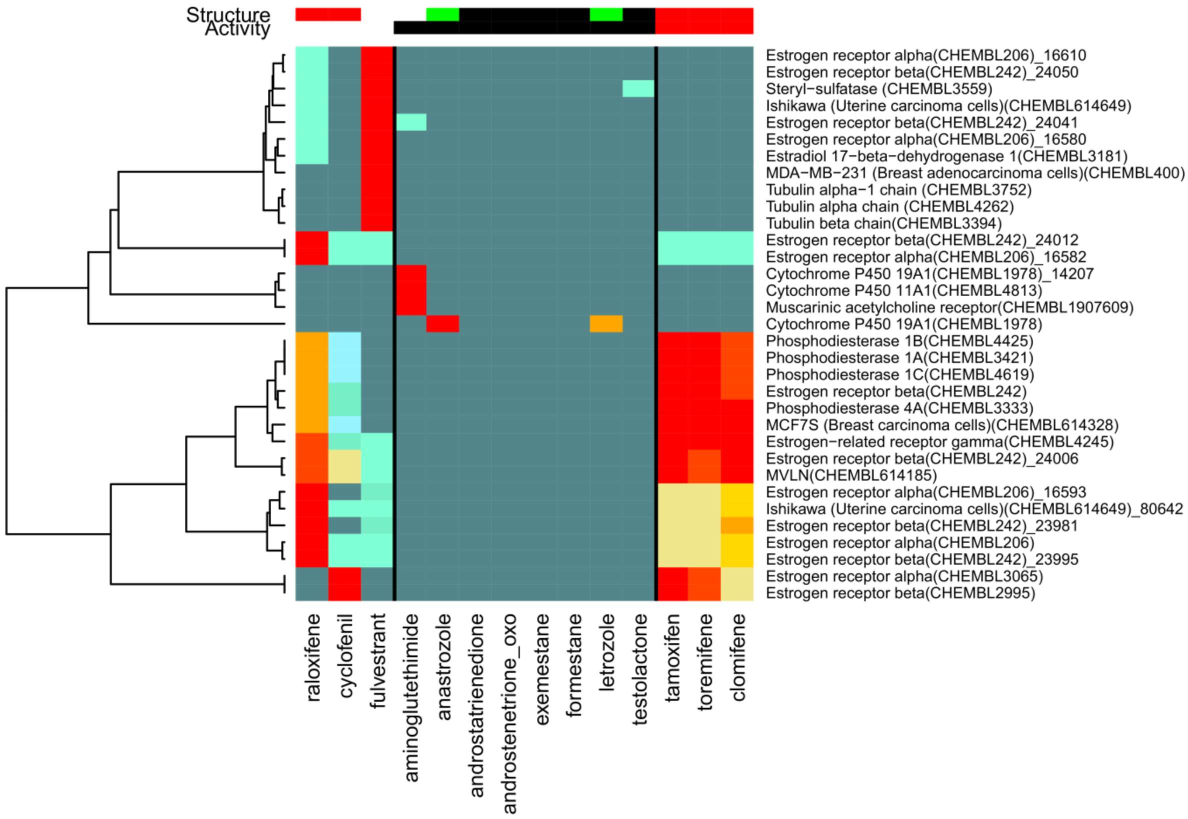

Figure S4A. The predicted molecule-target associations obtained by querying the 11 explicitly prohibited S4 Hormone Antagonists and Modulators against our refined families derived from ChEMBL.

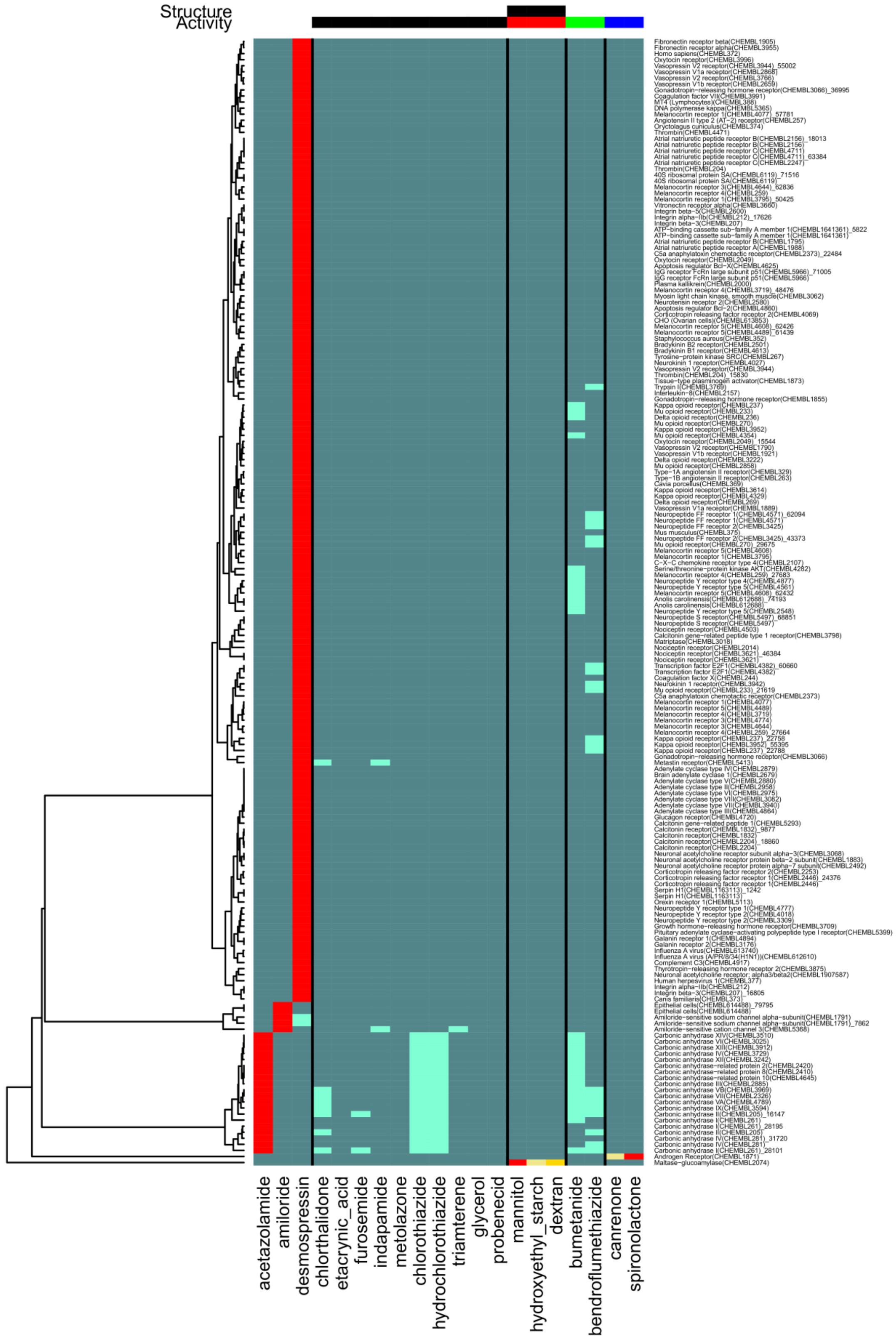

Figure S5A. The predicted molecule-target associations obtained by querying the 11 explicitly prohibited S5 Diuretics and Masking Agents against our refined families derived from ChEMBL.

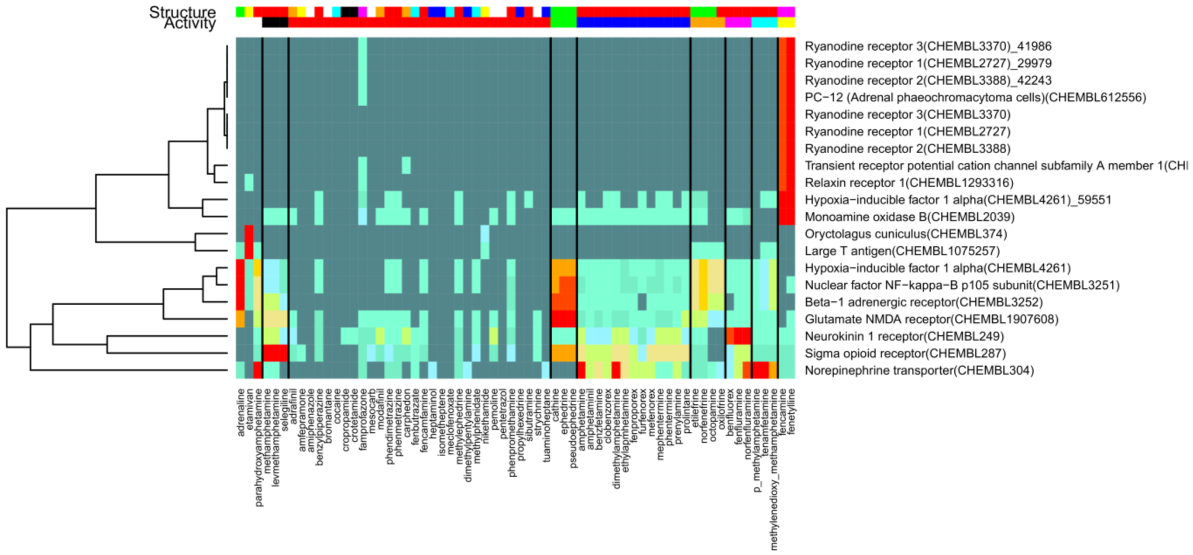

Figure S6A. The predicted molecule-target associations obtained by querying the 11 explicitly prohibited S6 Stimulants against our refined families derived from ChEMBL.

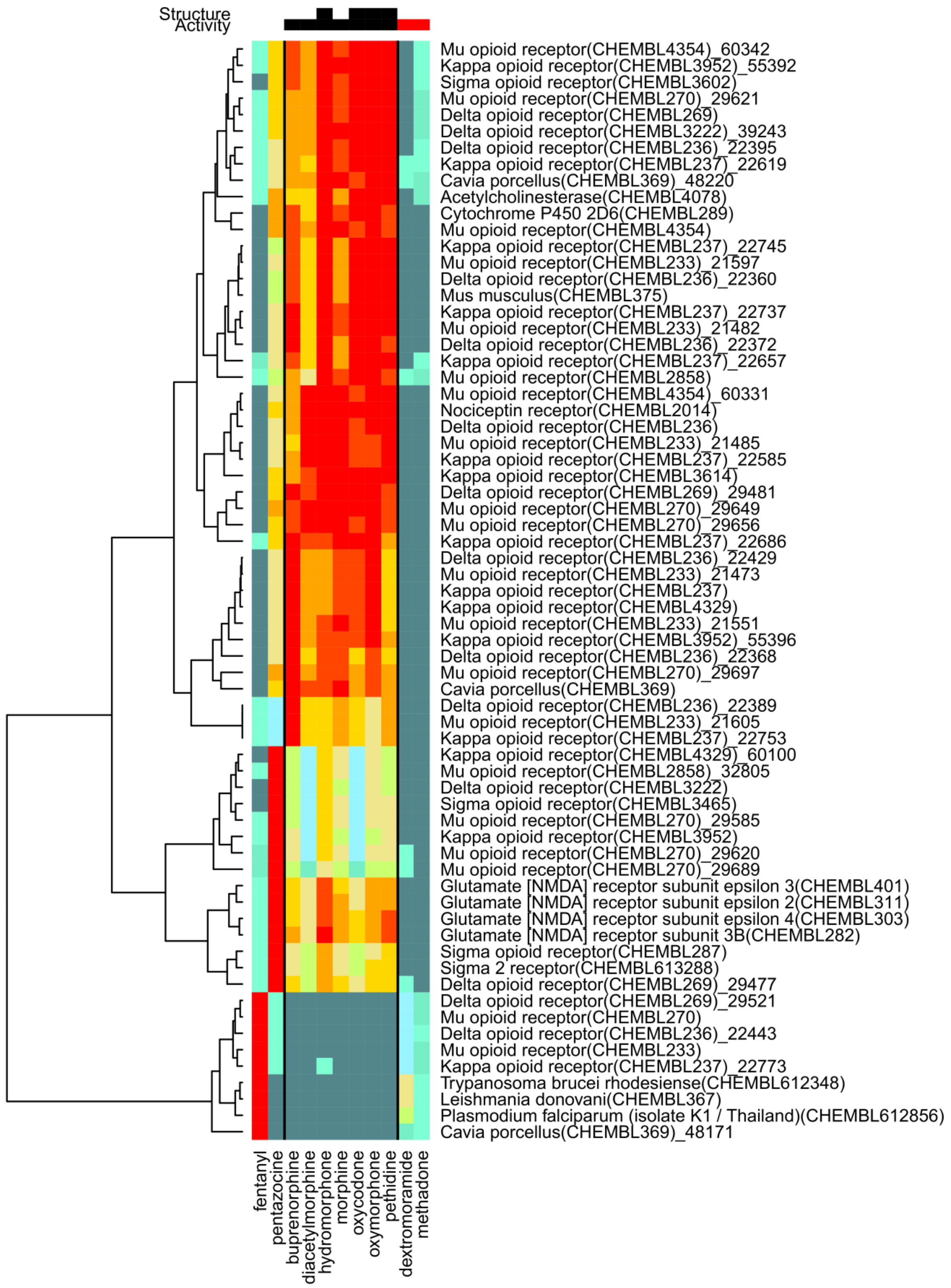

Figure S7A. The predicted molecule-target associations obtained by querying the 11 explicitly prohibited S7 Narcotics molecules against our refined families derived from ChEMBL.
